# Identifying and correcting for misspecifications in GWAS summary statistics and polygenic scores

**DOI:** 10.1016/j.xhgg.2022.100136

**Published:** 2022-08-18

**Authors:** Florian Privé, Julyan Arbel, Hugues Aschard, Bjarni J. Vilhjálmsson

**Affiliations:** 1 National Centre for Register-Based Research, Aarhus University, 8210 Aarhus, Denmark; 2 Université Grenoble Alpes, Inria, CNRS, Grenoble INP, LJK, 38000 Grenoble, France; 3 Department of Computational Biology, Institut Pasteur, Université Paris Cité, 75015 Paris, France; 4 Program in Genetic Epidemiology and Statistical Genetics, Harvard T.H. Chan School of Public Health, Boston, MA 02115, USA; 5 Bioinformatics Research Centre, Aarhus University, 8000 Aarhus, Denmark

**Keywords:** GWAS summary statistics, misspecifications, polygenic scores

## Abstract

Publicly available genome-wide association studies (GWAS) summary statistics exhibit uneven quality, which can impact the validity of follow-up analyses. First, we present an overview of possible misspecifications that come with GWAS summary statistics. Then, in both simulations and real-data analyses, we show that additional information such as imputation INFO scores, allele frequencies, and per-variant sample sizes in GWAS summary statistics can be used to detect possible issues and correct for misspecifications in the GWAS summary statistics. One important motivation for us is to improve the predictive performance of polygenic scores built from these summary statistics. Unfortunately, owing to the lack of reporting standards for GWAS summary statistics, this additional information is not systematically reported. We also show that using well-matched linkage disequilibrium (LD) references can improve model fit and translate into more accurate prediction. Finally, we discuss how to make polygenic score methods such as lassosum and LDpred2 more robust to these misspecifications to improve their predictive power.

## Teaser

This work studies the impact of misspecifications in summary statistics from genome-wide association studies. It provides an overview of such misspecifications, how they can arise, and what negative consequences they can have on follow-up analyses. It also investigates possible corrections, with the main goal of improving polygenic scores.

## Introduction

Contrary to individual-level genotypes and phenotypes, summary statistics resulting from genome-wide association studies (GWAS) are widely available, and very large sample sizes can be obtained through meta-analyses.^1^ GWAS summary statistics have been extensively used to derive polygenic scores (PGS), perform fine-mapping, and estimate a range of key genetic architecture parameters.^2^, ^3^, ^4^ However, GWAS summary statistics come with uneven imputation accuracy. There is also heterogeneity in the information made available in these summary statistics; for example, per-variant imputation INFO scores, sample sizes, and allele frequencies are often missing. Moreover, many methods based on summary statistics use Bayesian models and iterative algorithms, which can be particularly sensitive to model misspecifications.^5^^,^^6^

We present an overview of possible misspecifications that come with GWAS summary statistics in Table 2. First, the total sample size used can be misestimated, which would result in a biased estimation of the SNP heritability. For example, the total effective sample size is overestimated when computed from the total number of cases and controls from a meta-analysis of binary outcomes;^7^ using BOLT-LMM summary statistics can result in an increased effective sample size;^8^^,^^9^ and using SAIGE on binary traits with a large prevalence can result in a reduced effective sample size.^10^^,^^11^ Second, per-variant sample sizes can vary substantially and be much smaller than the total sample size when meta-analyzing GWAS summary statistics from multiple cohorts with different sets of variants.^12^ Using very different per-variant sample sizes can be problematic for models implicitly assuming that summary statistics have all been derived from the same individuals.^13^^,^^14^ Third, many genetic variants are imputed, with association statistics being reported for the allele dosages instead of the true alleles, which can lead to some bias in the summary statistics. Fourth, the imputation quality of each variant (e.g., INFO scores), often used in a quality control (QC) step in statistical genetics analyses, can be severely misestimated (often overestimated) when computed from multi-ancestry individuals instead of a more homogeneous subset. Fifth, errors such as allele inversions can be present in the summary statistics; QC is particularly important here. Sixth, many follow-up analyses require using linkage disequilibrium (LD) from a reference panel. There can be some mismatch between the GWAS summary statistics and the LD reference used, which can, e.g., lead to suboptimal predictive performance for polygenic scores.

Here we investigate some of these misspecifications and propose adjustments to improve the predictive performance of polygenic scores derived from GWAS summary statistics. We approach this from three different angles. First, based on additional summary information such as the imputation INFO scores and allele frequencies from the GWAS summary statistics, we refine our previously proposed QC.^15^ This QC consists in comparing standard deviations (of genotypes) inferred from GWAS summary statistics with the ones computed from a reference panel. This is useful to check that the input parameters used are consistent with one another. This was particularly important for LDpred2-auto, which directly estimates two key model parameters, the SNP heritability and polygenicity, from the data.^15^ Here we further show that standard deviations of imputed genotypes (allele dosages) are lower than the expected values under Hardy-Weinberg equilibrium. Second, we investigate possible adjustments to apply to the input parameters of PGS methods using summary statistics, namely the reference linkage disequilibrium (LD) matrix, the GWAS effect sizes, their standard errors, and their corresponding sample sizes. For example, we show that GWAS effect sizes computed from imputed dosages are larger in magnitude than when computed from true genotypes. Third, we introduce two new optional parameters in LDpred2-auto to make it more robust to these types of misspecification. Note that, in this paper, we use “robust” to mean that we obtain predictive performance similar to that when there is no misspecification. We also reimplement and use a new version of lassosum, called lassosum2, in which we better handle when per-variant sample sizes are different. We focus our investigations on LDpred2 and lassosum2 for two reasons. First, multiple studies have shown that LDpred2 and lassosum rank among the best methods for single-trait polygenic prediction.^15^, ^16^, ^17^, ^18^ Second, lassosum2 now uses the exact same input parameters as LDpred2, which makes it easy for us to test the different QCs and adjustments presented here. We additionally investigate PRS-CS and SBayesR, two competitive PGS methods,^19^^,^^20^ for which we develop some code to convert between the different formats required for the LD matrices and input GWAS summary statistics.

## Material and methods

### Data for simulations

We use the UK Biobank imputed (BGEN) data.^21^ We restrict individuals to the ones used for computing the principal components (PCs) in the UK Biobank (field 22020). These individuals are unrelated and have passed some QC including removing samples with a missing rate on autosomes larger than 0.02, having a mismatch between inferred sex and self-reported sex, and outliers based on heterozygosity (more details can be found in section S3 of Bycroft et al.^21^). To obtain a set of genetically homogeneous individuals, we compute a robust Mahalanobis distance based on the first 16 PCs and further restrict individuals to those within a log-distance of 5.^22^ This results in 362,307 individuals of Northwestern European ancestry. We randomly sample 300,000 individuals to form a training set (e.g., to run the GWAS), 10,000 individuals to form a validation set (to tune hyper-parameters), and use the remaining 52,307 individuals to form a test set (to evaluate final predictive models).

Among genetic variants on chromosome 22 and with a minor allele frequency larger than 0.01 and an imputation INFO score larger than 0.4 (as reported by the UK Biobank), we sample 40,000 of them according to the inverse of the INFO score density so that they have varying levels of imputation accuracy (Figure S1). We read the UK Biobank data into two different datasets using function snp_readBGEN from R package bigsnpr,^23^ one by reading the BGEN data as hard-called genotypes by randomly sampling according to the imputation probabilities (of being 0, 1, or 2), and another one by reading it as dosages (i.e., expected values according to the imputation probabilities). The first dataset is used as what could be the real genotype calls and the second dataset as what would be its imputed version; this design technique was used in Privé et al.^24^

### Data for real analyses

We also use the UK Biobank data for validation/testing in real-data analyses, and use the same individuals of Northwestern European ancestry as described in the previous section. We sample 10,000 individuals to form a validation set and use the remaining 352,307 individuals as a test set. We restrict to the 1,054,315 HapMap3 variants used in the LD reference provided in Privé et al.^15^

To define phenotypes in the UK Biobank, we first map ICD10 and ICD9 codes (UK Biobank fields 40001, 40002, 40006, 40013, 41202, 41270, and 41271) to phecodes using R package PheWAS.^25^^,^^26^ We also use some continuous phenotypes, namely vitamin D (data-field 30890), height (50), body mass index (BMI) (21001), systolic blood pressure (4080), and high-density lipoprotein (HDL) cholesterol (30760).

To derive polygenic scores, we use published GWAS summary statistics listed in Table 1. We also use GWAS summary statistics for five disease endpoints from FinnGen^33^ (release 6) and for four continuous outcomes from Biobank Japan.^34^

**Table 1:** Summary of external GWAS summary statistics used

Trait	GWAS citation	Effective GWAS sample size	No. of GWAS variants	No. of matched variants with INFO > 0.4	Mean INFO
Breast cancer (BrCa) (iCOGS)	Michailidou et al.^27^	87,037	11,792,542	1,051,242	0.841
Breast cancer (BrCa) (OncoArray)	Michailidou et al.^27^	104,442	11,792,542	1,054,233	0.968
Type 1 diabetes (T1D) (Affymetrix)	Censin et al.^28^	5516	8,996,866	934,712	0.885
Type 1 diabetes (T1D) (Illumina)	Censin et al.^28^	7982	8,996,866	949,334	0.942
Prostate cancer (PrCa)	Schumacher et al.^29^	135,316	20,370,946	818,400	0.969
Depression (MDD) (without UK Biobank)	Wray et al.^30^	110,464	9,874,289	1,049,455	0.968
Coronary artery disease (CAD)	Nikpay et al.^31^	129,014	9,455,778	1,052,200	0.982
Vitamin D	Jiang et al.^32^	79,366	2,579,296	1,016,935	N/A

PrCa summary statistics have many variants with a missing INFO score, which we discard. We also restrict to variants with an INFO score larger than 0.4. Note that vitamin D summary statistics do not report INFO scores.

### Model for summary statistics

In this paper, we extensively use the following formula:

(Equation 1) $$S_{j}=\text{sd}\left(G_{j}\right)\approx \frac{\text{sd}\left(y\right)}{\sqrt{n_{j}\;\text{se}{\left({\hat{\gamma }}_{j}\right)}^{2}+{\hat{\gamma }}_{j}^{2}}}\;,$$

where ${\hat{\gamma }}_{j}$ is the marginal GWAS effect size of variant j, $n_{j}$ is the GWAS sample size associated with variant j, y is the vector of phenotypes, and $G_{j}$ is the vector of genotypes for variant j. This formula is used in LDpred2.^15^^,^^35^ Note that, for a binary trait for which logistic regression is used, we have instead

(Equation 2) $$\text{sd}\left(G_{j}\right)\approx \frac{2}{\sqrt{n_{j}^{\text{eff}}\;\text{se}{\left({\hat{\gamma }}_{j}\right)}^{2}+{\hat{\gamma }}_{j}^{2}}}\;,$$

where $n_{j}^{\text{eff}}=\frac{4}{1/n_{j}^{\text{cases}}+1/n_{j}^{\text{controls}}}$.

It is assumed that the marginal (GWAS) effects follow

(Equation 3) $$\begin{array}{ll} \hat{\gamma }|S,R,\gamma & ∼N\left(S^{-1}RS\gamma ,S^{-1}RS^{-1}\right),\;\text{or},\\ S\hat{\gamma }|S,R,\gamma & ∼N\left(RS\gamma ,R\right), \end{array}$$

where R is the correlation matrix of genotypes.^13^ Then PGS methods aim at inferring γ, the true causal (per-allele) effects, from S, R, and $\hat{\gamma }$. However, in practice, we only have estimates for S and R, which causes some model misspecifications. Moreover, ubiquitous correlations between the large number of variants may cause a Gibbs sampler or any iterative approach used in PGS methods to fail in properly estimating genetic effect sizes.^36^

### GWAS sample size imputation

We can impute $n_{j}$ from Equation (1) using

(Equation 4) $$n_{j}\approx \frac{\text{var}\left(y\right)/\text{var}\left(G_{j}\right)-{\hat{\gamma }}_{j}^{2}}{\text{se}{\left({\hat{\gamma }}_{j}\right)}^{2}}\;,$$

and impute $n_{j}^{\text{eff}}$ from Equation (2) using

(Equation 5) $$n_{j}^{\text{eff}}\approx \frac{4/\text{var}\left(G_{j}\right)-{\hat{\gamma }}_{j}^{2}}{\text{se}{\left({\hat{\gamma }}_{j}\right)}^{2}}.$$

In practice, we also bound these estimates to be between 0.5⋅N and 1.1⋅N, where N is the total (effective) sample size. Also note that the estimate of $\text{var}\left(G_{j}\right)$ has to account for the imputation accuracy, i.e., use $2\cdot f_{j}\cdot \left(1-f_{j}\right)\cdot {\text{INFO}}_{j}$ (section “misspecification when using imputed allele dosages”). In Equations 1 and 4, we estimate var(y) by the first percentile of $0.5\left(N\;\text{se}{\left({\hat{\gamma }}_{j}\right)}^{2}+{\hat{\gamma }}_{j}^{2}\right)$, where the first percentile approximates the minimum and is robust to outliers.

### New implementation of lassosum in bigsnpr

Instead of using a regularized version of the correlation matrix R parameterized by s, $R_{s}=\left(1-s\right)R+sI$ (where 0<s≤1), we use $R_{\delta }=R+\delta I$ (where δ>0), which makes it clearer that lassosum is also using L2-regularization (therefore elastic-net). Then, from Mak et al.,^16^ the solution from lassosum can be obtained by iteratively updating

$$\beta_{j}^{\left(t\right)}=\left\{ \begin{array}{cc} \frac{\text{sign}\left(u_{j}^{\left(t\right)}\right)\left(\left|u_{j}^{\left(t\right)}\right|-\lambda \right)}{{\tilde{X}}_{j}^{T}{\tilde{X}}_{j}+\delta } & \text{if }\left|u_{j}^{\left(t\right)}\right|>\lambda \;\\ 0 & \text{otherwise} \end{array}\right.$$

where

$$u_{j}^{\left(t\right)}=r_{j}-{\tilde{X}}_{j}^{T}\left(\tilde{X}\beta^{\left(t-1\right)}-{\tilde{X}}_{j}\beta_{j}^{\left(t-1\right)}\right)\;.$$

Following the notations from Privé et al.,^15^ denote $\tilde{X}=\frac{1}{\sqrt{n-1}}C_{n}GS^{-1}$, where G is the genotype matrix, $C_{n}$ is the centering matrix, and S is the diagonal matrix of standard deviations of the columns of G. Then ${\tilde{X}}_{j}^{T}\tilde{X}=R_{j,.}=R_{.,j}^{T}$ and ${\tilde{X}}_{j}^{T}{\tilde{X}}_{j}=1$. Moreover, using the notations from Privé et al.,^15^ $u_{j}^{\left(t\right)}={\hat{\beta }}_{j}-R_{.,j}^{T}\beta^{\left(t-1\right)}+\beta_{j}^{\left(t-1\right)}$, where $r_{j}={\hat{\beta }}_{j}=\frac{{\hat{\gamma }}_{j}}{\sqrt{n_{j}\;\text{se}{\left({\hat{\gamma }}_{j}\right)}^{2}+{\hat{\gamma }}_{j}^{2}}}$ and ${\hat{\gamma }}_{j}$ is the GWAS effect of variant j, and n is the GWAS sample size.^16^^,^^35^ Then computing $R_{.,j}^{T}\beta^{\left(t-1\right)}$ is the most time-consuming part of each iteration. To make this faster, instead of computing $R_{.,j}^{T}\beta^{\left(t-1\right)}$ at each iteration (j and t), we can start with an initial vector of 0s only (for all j) since $\beta^{\left(0\right)}\equiv 0$, then update this vector when $\beta_{j}^{\left(t\right)}\neq \beta_{j}^{\left(t-1\right)}$ only. Note that only positions k for which $R_{k,j}\neq 0$ must be updated in this vector $R_{.,j}^{T}\beta^{\left(t-1\right)}$. Since bigsnpr v1.10.4, we now also use this updating strategy in LDpred2 (-grid and -auto), which makes it much faster, especially when the polygenicity is small.

In this new implementation of the lassosum model, which we call lassosum2, the input parameters are the correlation matrix R, the GWAS summary statistics (${\hat{\gamma }}_{j}$, $\text{se}\left({\hat{\gamma }}_{j}\right)$, and $n_{j}$), and the two hyper-parameters λ and δ. Therefore, except for the two hyper-parameters, lassosum2 uses the exact same input parameters as LDpred2.^15^ We try δ∈{0.001,0.01,0.1,1} by default in lassosum2, instead of s∈{0.2,0.5,0.8,1.0} in lassosum. For λ, the default in lassosum uses a sequence of 20 values equally spaced on a log scale between 0.1 and 0.001. By default in lassosum2, we use a similar sequence of 30 values, but between $\lambda_{0}$ and $\lambda_{0}/100$ instead, where $\lambda_{0}=\max_{j}\left|{\hat{\beta }}_{j}\right|$ is the minimum λ for which no variable enters the model because the L1-regularization is too strong. To make lassosum2 more robust to different per-variant sample sizes $n_{j}$, we also introduce per-variant penalty factors $\sqrt{\max_{k}\left(n_{k}\right)/n_{j}}$, by which we internally multiply λ and δ to penalize variants with smaller GWAS sample sizes more. Note that we do not provide an “auto” version using pseudo-validation (as in lassosum^16^), since we have not found the pseudo-validation scores to be consistent enough with the predictive performance (Figure S2). Also note that, as in LDpred2, we run lassosum2 genome-wide using a sparse correlation matrix which assumes that variants further away than 3 cM are not correlated. Contrary to lassosum, we do not require splitting the genome into independent LD blocks any longer, yet we recommend to do so for robustness in LDpred2 and for extra speed gain (cf. section “new LD reference”).

### LDpred2-low-h2 and LDpred2-auto-rob

Here we introduce the small changes made to LDpred2 (-grid and -auto) in order to make them more robust. First, LDpred2-low-h2 simply consists in running LDpred2-grid while testing $h^{2}$ within $\left\{0.3,0.7,1,1.4\right\}\cdot h_{\text{LDSC}}^{2}$ (note the added 0.3 compared with Privé et al.^15^), where $h_{\text{LDSC}}^{2}$ is the heritability estimate from LD score regression. Indeed, we show in simulations here that using lower values for $h^{2}$ may provide higher predictive performance in the case of misspecifications (thanks to more shrinkage of the effects). In simulations, because of the large misspecifications, we use a larger grid over $\left\{0.01,0.1,0.3,0.7,1,1.4\right\}\cdot h_{\text{LDSC}}^{2}$.

For LDpred2-auto, we introduce two new parameters. The first one, shrink_corr, allows for shrinking off-diagonal elements of the correlation matrix. This is similar to parameter “s” in lassosum (section “new implementation of lassosum in bigsnpr”), and acts as a means of regularization. We use a value of 0.9 in simulations and 0.95 in real data when running “LDpred2-auto-rob” here, and the default value of 1 (with no effect) when running “LDpred2-auto.” The second new parameter, allow_jump_sign, controls whether variant effect sizes can change sign over two consecutive iterations of the Gibbs sampler. When setting this parameter to false (in the method we refer to as “LDpred2-auto-rob” here), this forces the effects to go through 0 before changing sign. This is useful to prevent instability (oscillation and ultimately divergence) of the Gibbs sampler under large misspecifications, and is also useful for accelerating convergence of chains with a large initial value for p (the proportion of causal variants).

### New LD reference

We form nearly independent LD blocks using the optimal algorithm developed in Privé.^37^ Note that a new parameter max_r2 has now been added to this method, which controls the maximum single squared correlation allowed outside blocks and offers an extra guarantee that the splitting is good and makes the function much faster by discarding many possible splits. For different numbers of blocks and maximum number of variants in each block, we use the split that minimizes $\left(C2\cdot \sqrt{5+C1}\right)$, where C1 is the sum of squared correlations outside the blocks and C2 is the sum of squared sizes of the blocks (Figure S3). Twelve blocks from chromosome 22 are used in the simulations, and between 731 and 2,527 in the real-data analyses (cf. next section). Having a correlation matrix with independent blocks prevents small errors in the algorithm (e.g., the Gibbs sampler in LDpred2) from propagating to too many variants. It also makes running LDpred2 (and lassosum2) faster, taking e.g., 70% of the initial time (since only 70% of the initial non-zero values of the correlation matrix are kept). Note that we also compute the correlations within the same blocks and from the same data for PRS-CS.

We have also developed a new “compact” format for the SFBMs (sparse matrices on disk). Instead of using something similar to the standard “compressed sparse column” format which stores all {i,x(i,j)} for a given column j, we only store the first index $i_{0}$ and all the contiguous values $\left\{x\left(i_{0},j\right),\;x\left(i_{0}+1,j\right),\;\ldots \right\}$ up to the last non-zero value for this column j. This makes this format about twice as efficient for both LDpred2 and lassosum2 (in terms of both memory and speed). Therefore, using both this new format and the LD blocks should divide computation times by 3 (without counting the faster updating strategy of residuals).

### Alternative LD reference for FinnGen and Biobank Japan

To be used with GWAS summary statistics from FinnGen, we investigate three different LD reference panels. To define homogeneous ancestry groups in the UK Biobank, we include all individuals within a specific distance to a population center in the PC analysis space.^35^^,^^38^ We use either the 503 European (including 99 Finnish) individuals from the 1000 Genomes (1000G) data,^39^ 404 Finnish-like UK Biobank plus the 99 Finnish 1000G individuals (also 503 in total), or the 10,000 UK individuals from the validation set we use in this paper. 1,454, 731, and 1,165 independent LD blocks are identified for these three LD references, respectively.

To be used with GWAS summary statistics from Biobank Japan, we also investigate three different LD reference panels. We use either the 504 East Asian (including 104 Japanese) individuals from the 1000G, 400 Japanese-like UK Biobank plus the 104 Japanese 1000G individuals (also 504 in total), or 2,041 East Asian UK Biobank individuals (including the 400 previous Japanese individuals). For the small LD references (based on ∼500 individuals), we restrict to variants with a minor allele frequency (MAF) >0.02. For the one based on 2,041 individuals, we restrict to MAF >0.01. We also construct versions of these LD references with independent LD blocks, as described in the previous section. 2,527, 1,769, and 2,196 independent LD blocks are identified for these three LD references, respectively.

## Results

### Misspecification of per-variant GWAS sample sizes

**Figure 1:**
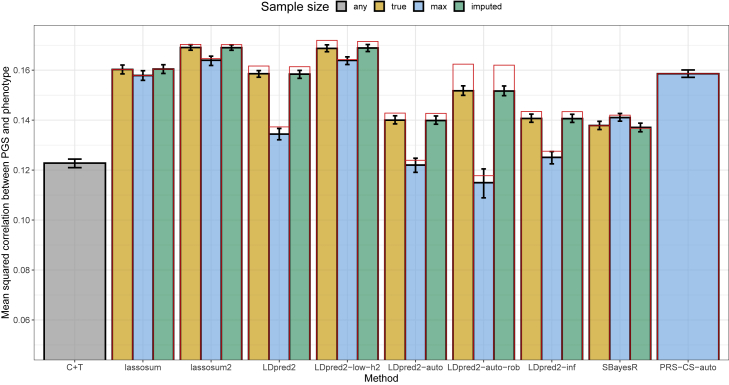
Results for the simulations with sample size misspecification, averaged over ten simulations for each scenario Reported 95% confidence intervals are computed from 10,000 non-parametric bootstrap replicates of the mean. The GWAS sample size is either “true” when providing the true per-variant sample size, “max” when providing instead the maximum sample size as a unique value to be used for all variants, “imputed” (cf. Equation 4), or “any” when the method does not use this information (the case for C+T). Red bars correspond to using the LD with independent blocks (section “new LD reference”), which is a requirement for lassosum and PRS-CS.

We design GWAS simulations where variants have different sample sizes, which is often the case when meta-analyzing GWAS summary statistics from multiple cohorts with different sets of variants.^12^ Using 40,000 variants from chromosome 22 (see material and methods), we simulate quantitative phenotypes by picking 2,000 causal variants at random, sampling their genetic effects (after scaling of the genotypes) from a normal distribution, and adding some Gaussian noise to the genetic component of the phenotype so that it has a heritability of 20%. This is implemented in function snp_simuPheno of R package bigsnpr.^23^ We then randomly divide the 40,000 variants into three groups: for half of the variants, we use 100% of 300,000 individuals for GWAS, but use only 80% for one quarter of the variants and 60% for the remaining quarter. We use a simple linear regression for GWAS, as implemented in function big_univLinReg of R package bigstatsr.^23^ We then run C+T, lassosum, lassosum2 (section “new implementation of lassosum in bigsnpr”), LDpred2-inf, LDpred2(-grid), LDpred2-auto, SBayesR (GCTB v2.03 with the automatic robust option), and PRS-CS-auto^15^^,^^16^^,^^19^^,^^20^^,^^24^ by using either the true per-variant GWAS sample sizes, the total sample size for all variants, or per-variant sample sizes imputed using Equation (4). When providing true per-variant GWAS sample sizes, squared correlations between the polygenic scores and the simulated phenotypes are of 0.123 for C+T, 0.160 for lassosum, 0.169 for lassosum2, 0.159 for LDpred2(-grid), 0.140 for LDpred2-auto, 0.141 for LDpred2-inf, 0.138 for SBayesR, and 0.159 for PRS-CS-auto (Figure 1, averaging over ten simulations). Results when using imputed sample sizes are very similar to when using the true ones. Note that C+T does not use this sample size information, and that PRS-CS can only be provided with a single value (we use the maximum one). When using the total GWAS sample size instead of the per-variant sample sizes, predictive performance slightly decreases to 0.158 for lassosum and to 0.164 for lassosum2, but dramatically decreases for LDpred2 with new values of 0.134 for LDpred2-grid, 0.122 for LDpred2-auto, and 0.125 for LDpred2-inf (Figure 1). This extreme simulation scenario shows that LDpred2 can be sensitive to the misspecification of per-variant GWAS sample sizes, whereas lassosum (and lassosum2) seems little affected by this. With 0.141 for SBayesR, predictive performance is actually larger than when using the true per-variant sample sizes.

We conduct further investigations to explain the results of Figure 1. First, the results for LDpred2-auto and SBayesR are similar to LDpred2-inf in these simulations because the internal polygenicity estimate p (proportion of causal variants) of LDpred2-auto, and similarly the proportion of non-null variants in SBayesR, always continuously increases up to 1 in the Gibbs sampler, which makes them behave as an infinitesimal model (p=1). To overcome this limitation in LDpred2-auto, we introduce two new parameters to make it more robust, and refer to this as “LDpred2-auto-rob” here (section “LDpred2-low-h2 and LDpred2-auto-rob”). The first parameter prevents p from diverging to 1 while the second shrinks the off-diagonal elements of the LD matrix (a form of regularization). Second, for lassosum2, results for a grid of parameters (over λ and δ) are quite smooth compared with LDpred2 (Figures S4 and S5). In these simulations with misspecified per-variant sample sizes, it seems highly beneficial to use a small value for the SNP heritability hyper-parameter $h^{2}$ in LDpred2, e.g., a value of 0.02 or even 0.002 when the true value is 0.2 (Figure S5). Indeed, using a small value for this hyper-parameter induces a larger regularization (shrinkage) on the effect sizes. Here we call “LDpred2-low-h2” when running LDpred2(-grid) with a grid of hyper-parameters including these low values for $h^{2}$. Results with LDpred2-low-h2 improve from 0.159 to 0.169 when using true sample sizes and from 0.134 to 0.164 when using the maximum sample size (Figure 1). Finally, we introduce here a last change for robustness: we form independent LD blocks in the LD matrix to prevent small errors in the Gibbs sampler to propagate to too many variants (section “new LD reference”). This change seems to solve convergence issues of LDpred2 in these simulations (Figure S5) and further improves predictive performance for all LDpred2 methods (Figure 1).

As secondary analysis, we rerun these simulations with a smaller heritability of 4% instead. While predictive performance are overall much lower, relative performance are less attenuated when using the maximum sample size, otherwise results are highly consistent as before (Figure S6). We also try using the shrunk LD matrix from GCTB that is used, e.g., for SBayesR;^20^ in these simulations, higher predictive performance is obtained for LDpred2-auto with this shrunken LD matrix, whereas SBayesR benefits from using LDpred2’s windowed LD matrix (Figure S7).

### Misspecification when using imputed allele dosages

Marchini and Howie^40^ showed that the IMPUTE INFO measure is highly concordant with the MACH measure $\frac{\text{var}\left(G_{j}\right)}{2{\hat{\theta }}_{j}\left(1-{\hat{\theta }}_{j}\right)}$, where ${\hat{\theta }}_{j}$ is the estimated allele frequency of $G_{j}$, the genotypes for variant j. Therefore, when using the expected genotypes from imputation (allele dosages), their standard deviations are often lower than the expected value under Hardy-Weinberg equilibrium, because ${\text{INFO}}_{j}\approx \frac{\text{var}\left(G_{j}\right)}{2{\hat{\theta }}_{j}\left(1-{\hat{\theta }}_{j}\right)}$. In simulations (cf. section “data for simulations”), we verify that $\text{sd}{\left(G_{j}\right)}^{\text{true}}\approx \text{sd}{\left(G_{j}\right)}^{\text{imp}}/\sqrt{{\text{INFO}}_{j}}$ (Figure S8). As a direct consequence of the lower standard deviation of dosages, we also show that GWAS effect sizes $\hat{\gamma }$ computed from imputed dosages are overestimated: ${\hat{\gamma }}_{j}^{\text{true}}\approx {\hat{\gamma }}_{j}^{\text{imp}}\cdot \sqrt{{\text{INFO}}_{j}}$ and $\text{se}{\left({\hat{\gamma }}_{j}\right)}^{\text{true}}\approx \text{se}{\left({\hat{\gamma }}_{j}\right)}^{\text{imp}}\cdot \sqrt{{\text{INFO}}_{j}}$ (Figures S9 and S10). This is the first correction of summary statistics we consider in the simulations below. As a second option, instead of using dosages to compute the GWAS summary statistics, it has been argued that using multiple imputation (MI) would be more appropriate.^41^ Here MI consists in forming multiple (e.g., 20) datasets of hard-called genotypes by sampling them according to the imputation probabilities stored in the BGEN files, performing a GWAS on each of these datasets, and pooling the GWAS estimates. In simulations, we show that ${\hat{\gamma }}_{j}^{\text{MI}}\approx {\hat{\gamma }}_{j}^{\text{imp}}\cdot {\text{INFO}}_{j}$ and $Z_{j}^{\text{MI}}\approx Z_{j}^{\text{imp}}\cdot {\text{INFO}}_{j}$, where $Z=\hat{\gamma }/\text{se}\left(\hat{\gamma }\right)$ (Figure S11). This is the second correction of summary statistics we implement in the simulations below, along with $n_{j}\cdot {\text{INFO}}_{j}$ as the new per-variant sample sizes. Finally, we consider an in-between solution as a third correction, using ${\hat{\gamma }}_{j}={\hat{\gamma }}_{j}^{\text{imp}}\cdot {\text{INFO}}_{j}$, $\text{se}\left({\hat{\gamma }}_{j}\right)=\text{se}{\left({\hat{\gamma }}_{j}\right)}^{\text{imp}}\cdot \sqrt{{\text{INFO}}_{j}}$, and $n_{j}\cdot {\text{INFO}}_{j}$ as the per-variant sample sizes. Note that we have recomputed INFO scores for the subset of 362,307 European individuals used in this paper, since they can differ substantially from the ones reported by the UK Biobank for the whole data (Figures S12 and S13).

**Figure 2:**
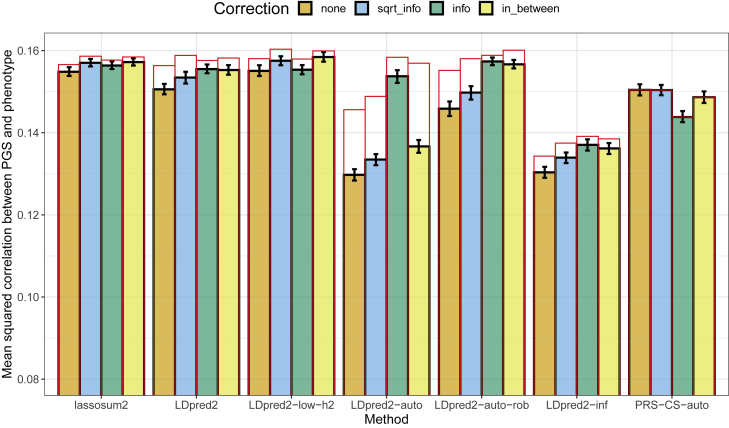
Results of predictive performance for the simulations using GWAS summary statistics from imputed dosage data, averaged over ten simulations for each scenario Reported 95% confidence intervals are computed from 10,000 non-parametric bootstrap replicates of the mean. Correction “sqrt_info” corresponds to using ${\hat{\gamma }}_{j}^{\text{imp}}\cdot \sqrt{{\text{INFO}}_{j}}$ and $\text{se}{\left({\hat{\gamma }}_{j}\right)}^{\text{imp}}\cdot \sqrt{{\text{INFO}}_{j}}$. Correction “info” corresponds to using ${\hat{\gamma }}_{j}^{\text{imp}}\cdot {\text{INFO}}_{j}$ and $n_{j}\cdot {\text{INFO}}_{j}$. Correction “in_between” corresponds to using ${\hat{\gamma }}_{j}^{\text{imp}}\cdot {\text{INFO}}_{j}$, $\text{se}{\left({\hat{\gamma }}_{j}\right)}^{\text{imp}}\cdot \sqrt{{\text{INFO}}_{j}}$, and $n_{j}\cdot {\text{INFO}}_{j}$. Red bars correspond to using the LD with independent blocks (section “new LD reference”), which is a requirement for PRS-CS.

To compare these three corrections, we conduct a simulation using the same 40,000 variants from chromosome 22 as before, whereby we simulate quantitative phenotypes assuming a heritability of 20% and 2,000 causal variants using the “true” dataset (cf. section “data for simulations”). We compute GWAS summary statistics from the dosage dataset and use these summary statistics to run LDpred2, lassosum2 and PRS-CS with either no correction of the summary statistics or with one of the three corrections described above. The LD references are computed from the validation set using the dataset with the “true” genotypes (i.e., same as before). For lassosum2 and LDpred2(-grid), which tune parameters using the validation set, correcting for imputation quality slightly improves predictive performance in these simulations (Figure 2). However, correcting for imputation quality can dramatically improve predictive performance for LDpred2-auto, provided the imputation quality is well estimated. Moreover, new additions for robustness introduced before, namely LDpred2-low-h2, LDpred2-auto-rob, and forming independent blocks in the LD matrix, also improve predictive performance for all corrections (Figure 2). However, these corrections do not prove beneficial for PRS-CS-auto (Figure 2).

When performing similar simulations with a heritability of 4% (instead of 20%), correction “sqrt_info” still provides slightly higher predictive performance for all methods (compared with no correction), yet the other two corrections sometimes provide lower predictive performance (Figure S14). In the real-data applications hereafter, we therefore choose to use the first correction, “sqrt_info,” which seems to more consistently provide better results and is simple because it is somewhat equivalent to post-processing PGS effects by multiplying them by $\sqrt{\text{INFO}}$.

### Mismatch between LD reference and GWAS summary statistics

**Figure 3:**
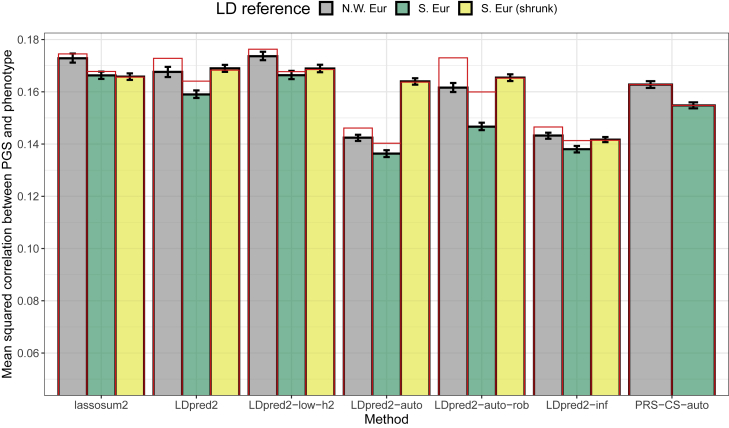
Results for the simulations with summary statistics with LD matrices based on two different populations One comes from the same ancestry used for computing the GWAS summary statistics (North-West Europe), while the other one comes from South Europe (alternative LD reference). Reported 95% confidence intervals are computed from 10,000 non-parametric bootstrap replicates of the mean. Red bars correspond to using the LD with independent blocks (section “new LD reference”), which is a requirement for PRS-CS.

Here we design simulations to understand the impact of using a mismatched LD reference panel, e.g., that comes from a different population compared with the one used to compute the GWAS summary statistics. We use the same simulation setup as before (see material and methods), i.e., using individuals of Northwestern European ancestry from the UK Biobank for training (GWAS), validation (tuning of hyper-parameters), and testing (the final models). In addition to the LD reference panel of Northwestern European ancestry, we design an alternative reference panel based on 10,000 individuals from South Europe by using the “Italy” center defined in Privé et al.^35^ Allele frequencies and pairwise correlations are quite similar between these two LD reference panels (Figure S15). When using this alternative LD reference panel instead of a well-matched one as in the previous sections, squared correlations between the polygenic scores and the simulated phenotypes drop from 0.173 to 0.166 for lassosum2, from 0.168 to 0.159 for LDpred2(-grid), from 0.174 to 0.169 for LDpred2-low-h2, from 0.142 to 0.136 for LDpred2-auto, from 0.162 to 0.147 for LDpred2-auto-rob, from 0.143 to 0.138 for LDpred2-inf, and from 0.163 to 0.155 for PRS-CS-auto (Figure 3, averaging over ten simulations). Forming independent LD blocks in these two LD matrices always improves predictive performance, yet again. We also compute and test using a shrunk LD matrix (from GCTB) for this alternative LD reference panel; again, using this type of LD matrix seems beneficial for LDpred2-auto. Finally, under the same simulation scenario but with a smaller simulated heritability of 4% (instead of 20%), there remains a small drop in predictive performance when using the alternative LD reference (Figure S16).

### Application to breast cancer summary statistics

**Figure 4:**
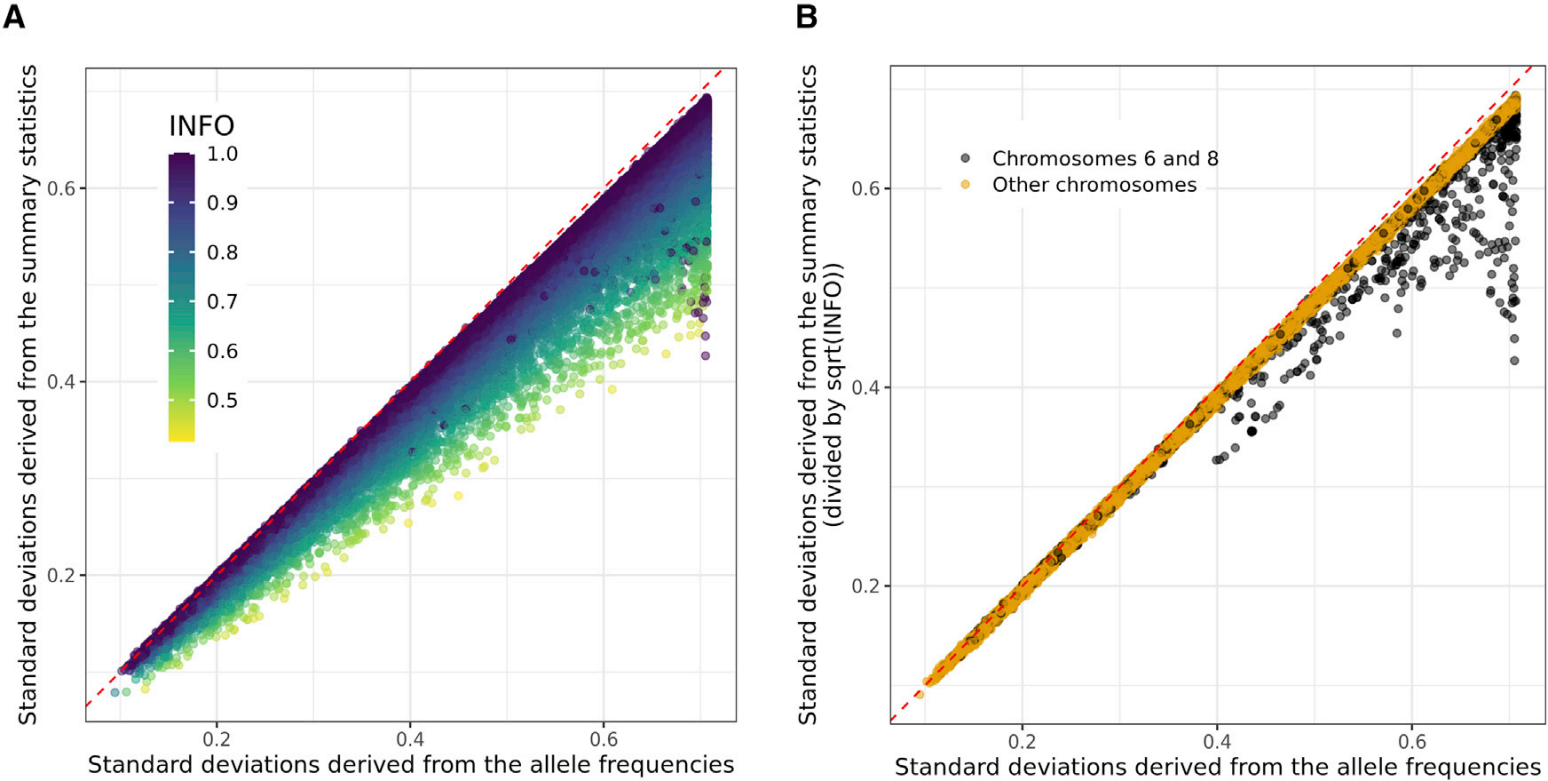
Comparison of standard deviations for quality control of GWAS summary statistics Standard deviations inferred from the OncoArray breast cancer GWAS summary statistics using Equation 2 (A: raw or B: dividing them by $\sqrt{\text{INFO}}$) versus the ones inferred from the reported GWAS allele frequencies $f_{j}$ (using $\sqrt{2f_{j}\left(1-f_{j}\right)}$). Only 100,000 HapMap3 variants are represented, at random.

In this section, we transition to using real data. Breast cancer GWAS summary statistics are interesting because they include results from two mega-analyses,^27^^,^^42^^,^^43^ which means that parameters reported in these GWAS summary statistics, such as INFO scores and sample sizes, are estimated with high precision. Imputation INFO scores for the OncoArray summary statistics are generally very good (mean of 0.968 after having restricted to HapMap3 variants, Figure S17) and better than the ones from iCOGS (mean of 0.841, Figure S18), probably because the iCOGS chip included around 200K variants only, compared with more than 500K variants for the OncoArray. For both summary statistics, we compare the standard deviations (of genotypes) inferred from the reported allele frequencies (i.e., $\sqrt{2f\left(1-f\right)}$ where f is the allele frequency, and denoted as sd_af) versus the ones inferred from the GWAS summary statistics (Equation 2, and denoted as sd_ss). As shown in Figures 4 and S19, there is a clear trend with sd_ss being lower than sd_af as INFO decreases; indeed, using $\text{sd}\_\text{ss/}\sqrt{\text{INFO}}$ provides a very good fit for sd_af, except for some variants of chromosomes 6 and 8 for the OncoArray summary statistics. Most of these outlier variants are either in region 25–33 Mbp of chromosome 6 or in 8–12 Mbp of chromosome 8 (Figure S20), which are two known long-range LD regions.^44^ We hypothesize that this is due to using PCs that capture LD structure instead of population structure, as covariates in the GWAS.^22^ To validate this hypothesis, we simulate a phenotype using HapMap3 variants of chromosome 6 for 10,000 individuals from the UK Biobank and then run GWAS with or without PC19 as covariate. PC19 from the UK Biobank was previously reported to capture LD structure in region 70–91 Mbp of chromosome 6.^22^ In these simulations, the same bias as in Figure 4B is observed for the variants in this region (Figure S21), confirming our hypothesis.

Therefore, providing an accurate imputation INFO score is useful for two reasons. First, it allows for correcting for a reduced standard deviation when using imputed data in the QC step we propose to better uncover possible problems with the GWAS summary statistics. Second, using one of the proposed corrections based on INFO scores may lead to an improved prediction when deriving polygenic scores. We apply these corrections to the two breast cancer summary statistics. In the comparison, we first use the QC proposed in Privé et al.^15^ (which ends up filtering on MAF here, which we call “qc1”). We then also filter out the two long-range LD regions of chromosomes 6 and 8 for the OncoArray summary statistics and remove around 500 variants when filtering on differences of allele frequencies (>0.1) between summary statistics and the validation dataset (“qc2”). As for the correction using INFO scores, we use the first correction, “sqrt_info,” which is simple because it is equivalent to post-processing PGS effects, by multiplying them by $\sqrt{\text{INFO}}$. Although results for both QC used are very similar, correcting using INFO scores slightly improves predictive performance when deriving polygenic scores based on iCOGS summary statistics (Figure S22). All other improvements introduced before have little to no effect here, probably because misspecifications are much smaller than in the simulations.

### Results for other phenotypes

**Figure 5:**
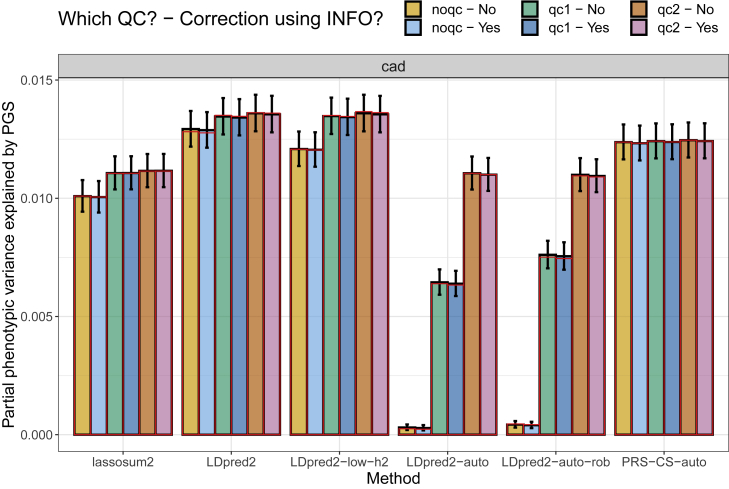
Variance explained of CAD in the UK Biobank by PGS derived from external summary statistics These are computed using function pcor of R package bigstatsr where 95% confidence intervals are obtained through Fisher’s Z-transformation; these values are then squared. Red bars correspond to using the LD with independent blocks (see material and methods), which is a requirement for PRS-CS.

We use other external GWAS summary statistics for which INFO scores are reported (see Table 1); they all have a very high mean INFO score (larger than 0.94), except for T1D-affy (0.885). QC plots comparing standard deviations usually show little deviation from the identity line (after the INFO score correction), except for coronary artery disease (CAD) summary statistics (Figures S23–S27). Note that the popular CAD summary statistics used here come from a multi-ancestry GWAS meta-analysis with more than 20% non-European samples.^31^^,^^38^ In Figures 5 and S28–S32, we then provide similar results as in Figure S22 for other phenotypes. For major depressive disorder (MDD) and prostate cancer (PrCa), most changes introduced before have little to no impact on predictive performance. For CAD and T1D, the QC proposed in Privé et al.^15^ and the additional QC proposed here provide much better predictive performance, especially for LDpred2-auto (and LDpred2-auto-rob) than when using no QC, showing how important this preliminary step is.

**Figure 6:**
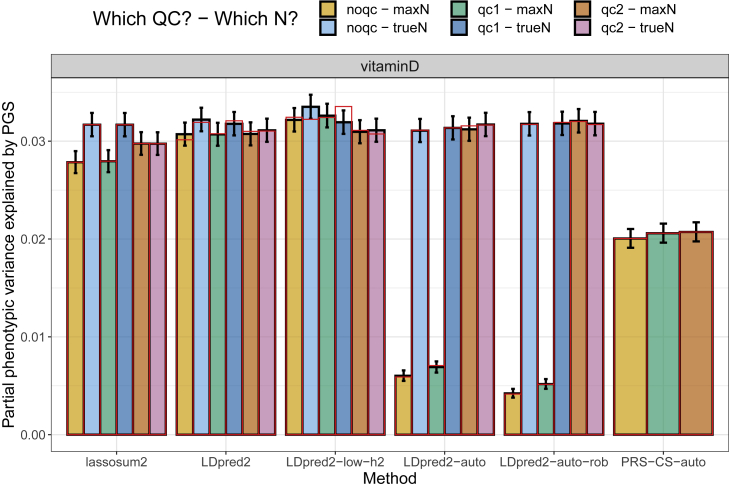
Variance explained of vitamin D in the UK Biobank by PGS derived from external summary statistics These are computed using function pcor of R package bigstatsr where 95% confidence intervals are obtained through Fisher’s Z-transformation; these values are then squared. Red bars correspond to using the LD with independent blocks (see material and methods), which is a requirement for PRS-CS.

We also investigate results for vitamin D GWAS summary statistics, which do not report INFO scores or allele frequencies, as opposed to previous ones. The QC procedure is thus less precise and uses allele frequencies from the LD reference (Figure S33). However, these summary statistics do report per-variant sample sizes, which cover a wide range of different values (Figure S34). Here we compare using either the maximum sample size or the true per-variant sample sizes when deriving polygenic scores, and an additional QC step, “qc2”, which refers to removing all variants with a per-variant sample size less than 70% of the maximum one. When the true per-variant sample sizes are used, the additional “qc2” does not seem to be necessary (Figure 6), which is a bit surprising to us. When the maximum sample size is used, this “qc2” is very important, especially for LDpred2-auto. Note that, when using the maximum sample size to derive “sd_ss” in “qc1” (not the case here; we used the per-variant sample sizes), “qc1” would remove variants with underestimated standard deviations due to overestimated sample sizes, which would effectively remove variants with low sample sizes (Figure S35), similar to the “qc2” used here.

For all phenotypes, PRS-CS-auto is very robust, whatever the QC procedure used (e.g., see the results for CAD, Figure 5). We believe this is because of the strong regularization used internally. We then run LDpred2-auto-rob for CAD with different shrinkage multiplicative coefficients for the off-diagonal elements of the LD matrix, from 1 (LD matrix unchanged) to 0 (using the identity matrix instead). When using a strong shrinkage of 0.3 or 0.4, results for LDpred2-auto-rob are similar regardless of the QC used (Figure S36), as previously observed for PRS-CS-auto. Results with LDpred2-auto-rob are always best when using “qc2”, the most stringent QC; then no shrinkage (1) seems needed, whereas a strong shrinkage leads to a drop in predictive performance. For phenotypes with relatively large genetic effects (e.g., vitamin D, Figure 6), PRS-CS-auto performs much worse, possibly due to using too much regularization.

### Application to FinnGen and Biobank Japan summary statistics

**Figure 7:**
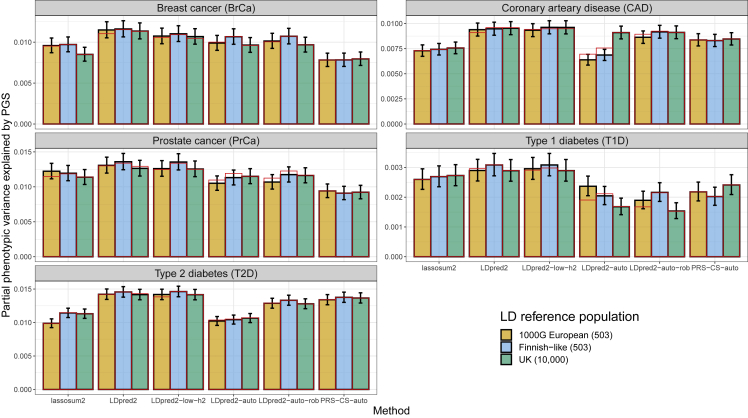
Results for PGS derived from five FinnGen GWAS summary statistics and using three different LD references Partial correlations are computed using function pcor of R package bigstatsr where 95% confidence intervals are obtained through Fisher’s Z-transformation, then all values are squared to report the phenotypic variance explained by PGS. Red bars correspond to using the LD with independent blocks (see material and methods), which is a requirement for PRS-CS.

Here we investigate the use of different LD reference panels with GWAS summary statistics from two large biobanks of isolated populations. We use GWAS summary statistics for five disease endpoints from FinnGen^33^ (release 6), namely breast and prostate cancers (BrCa and PrCa), CAD, and type 1 and type 2 diabetes (T1D and T2D). These were derived using SAIGE.^10^ First, for each phenotype, we estimate a global effective sample using the 80th percentile of imputed sample sizes from Equation 5. This estimation is only 84.7% for BrCa, 79.1% for PrCa, 73.1% for T1D, 64.5% for T2D, and 61.3% for CAD, when compared with the effective sample computed from the reported numbers of cases and controls. We believe this reduction in effective sample size is due to having related individuals included in the analyses as well as using SAIGE, as noted in the introduction. We then compare three different LD reference panels to use with these GWAS summary statistics (section “alternative LD reference for FinnGen and Biobank Japan”). Interestingly, using the small Finnish LD reference panel we have defined here, composed of only 503 individuals, seems to almost always provide more predictive polygenic scores than using the large UK panel composed of 10,000 individuals or the widely used European subset of the 1000G data (Figure 7). For PRS-CS-auto, none of the three LD references seems to consistently outperform the other two across all phenotypes considered. Note that the PGS here are validated in people of UK-like ancestry in the UK Biobank to obtain sufficient sample sizes.

We also use GWAS summary statistics for four continuous outcomes from Biobank Japan.^34^ These were derived using BOLT-LMM-inf.^8^ First, for each phenotype, we estimate a global power improvement provided by BOLT-LMM by comparing $\chi^{2}$ statistics (the boost of BOLT-LMM-inf versus linear regression) across genome-wide significant variants, as recommended in Loh et al.^8^ This estimated power ratio, which corresponds to the increase in effective sample size, is 1.143 for height, 1.039 for HDL cholesterol, 1.025 for BMI, and 1.013 for systolic blood pressure. We then compare three different LD reference panels to use with these GWAS summary statistics (section “alternative LD reference for FinnGen and Biobank Japan”). Interestingly, when looking at height and HDL cholesterol, where we could get the best predictive performance, using the smaller Japanese LD reference we have defined here seems to provide more predictive PGS than using the one using a larger set of East Asian individuals from the UK Biobank or the widely used East Asian subset of the 1000G data (Figure S37), except when using PRS-CS-auto to predict height. Note that the PGS here are validated in people of broad East Asian ancestry in the UK Biobank and for continuous outcomes to obtain moderate sample sizes for comparison.

### Recommendations

Based on previous results from simulations and real-data analyses, we use this section to suggest some recommendations on how to handle misspecifications in GWAS summary statistics and to get the most out of PGS. We recall that an overview of these misspecifications, along with possible consequences and possible corrections, is summarized in Table 2. First, standard QC should always be performed, e.g., variants with low frequency or low imputation quality should be removed; one must make sure to recompute MAF and INFO scores for the homogeneous subset of individuals of interest. Second, comparison of allele frequencies can be used to, e.g., detect problems with signs of effects while comparing standard deviations to detect other issues such as low per-variant GWAS sample sizes. Third, the LD reference panel should be as close as possible (in terms of genetic ancestry) to the data used to derive the GWAS. In case of any doubt, ancestry proportions can be estimated from GWAS allele frequencies using the method derived in Privé.^38^ Fourth, if some validation data is available for tuning hyper-parameters, one can use methods such as lassosum2 and LDpred2(-grid). The grid of parameters used in lassosum2 includes a broad range of regularization, which makes it very robust. For LDpred2, we recommend to also include a smaller value for the heritability in the grid of parameters to allow for more regularization when needed. As these two methods use the same data and the same principle (i.e., hyper-parameter tuning), one could run both methods and choose the best one when tuning hyper-parameters. Fifth, if no validation/tuning dataset is available, we recommend using LDpred2-auto with the two new parameters introduced here, or PRS-CS-auto if there are strong misspecifications and the phenotype of interest is known to have only small effects. Sixth, we recommend to form independent LD blocks in the LD matrix, as it proved to make the methods more robust (and slightly faster). Most of these new recommendations are included in the LDpred2 tutorial.

**Table 2:** Overview of possible misspecifications when using GWAS summary statistics, along with possible consequences and corrections

Misspecification	Possible consequence(s)	Possible correction
Misestimation of the total sample size^a^	bias estimation of SNP heritability^7^^,^^9^	estimation of the effective sample size
Differences in per-variant sample sizes	possible violation of model assumptions (e.g., Equation 3) resulting in poor predictive performance	imputation using Equations (Equation 4), (Equation 5), and filtering^b^
Using imputed dosages for GWAS	standard deviations of dosages are too small, resulting in overestimated effect sizes and standard errors	see section “misspecification when using imputed allele dosages”
Using imputed dosages for LD computation		none required; LD seems correctly estimated (Figure S38)
Misestimation of imputation INFO scores	misuse when filtering or adjusting input parameters based on these	recomputing from a homogeneous subset
Error in summary statistics (e.g., allele inversions)	strong violation of model assumptions, which can result in e.g., identifying false positives in fine-mapping^45^	quality control
Rounding of summary statistics (e.g., effect sizes and SEs)	add unnecessary noise to the data	none, but this has almost no impact on the predictive performance (Figure S39)
Ancestry mismatch between summary statistics and LD	strong mismatch of LD and allele frequencies, often resulting in divergence of models	checking ancestry proportions from GWAS summary statistics^38^
Any	possible violation of model assumptions resulting in poor predictive performance	quality control, using more regularization, and constraining LD to blocks

a Examples: using the total number of cases and controls in a meta-analysis of binary traits, a larger effective sample size when using BOLT-LMM summary statistics,^8^ and a reduced effective sample size when using SAIGE on binary traits with a large prevalence.^10^^,^^11^

b For example, removing SNPs with an effective sample size less than 0.67 times the 90th percentile of sample size.^46^

## Discussion

Here we have investigated misspecifications in GWAS summary statistics, focusing particularly on the impact of sample size heterogeneity and imputation quality, and the application to PGS methods. Previously, we proposed a QC based on comparing standard deviations (of genotypes) inferred from GWAS summary statistics with the ones computed from a reference panel.^15^ Here we show that we can refine this QC by deriving the latter directly from the reported allele frequencies in the GWAS summary statistics, and by correcting the former using imputation INFO scores. Using this refined QC, we are able to identify a potential issue with how PCs were derived in a GWAS of breast cancer. Fortunately, this has practically no effect on the predictive performance of the derived PGS. Additional QC can also be performed, e.g., comparing reported GWAS allele frequencies with the ones from the LD reference panel to detect genotyping errors or allele inversions. We perform this additional QC as part of “qc2” here. One can also run other QC tools such as DENTIST,^4^ and also infer ancestry proportions from summary statistics to make sure these are matching with the LD reference used.^38^

Note that, in this study, we mostly use GWAS summary statistics that include extended information (e.g., INFO scores and allele frequencies), yet most GWAS summary statistics do not provide such exhaustive information.^47^ We acknowledge that, in the case of a meta-analysis from multiple studies, providing a single INFO score per variant may not be possible. Solutions such as using a weighted averaged INFO score might be worth exploring in future studies. Nevertheless, this QC could be performed within each study before meta-analyzing results to ensure that resulting summary statistics have the best possible quality for follow-up analyses such as deriving PGS. Other information, the effective sample size per variant, is often missing from GWAS summary statistics. Sometimes it can even be challenging to recover the total effective sample size from large meta-analyses. We recall that when some studies have an imbalanced number of cases and controls, the total effective sample size of their meta-analysis should not be computed from the total numbers of cases and controls overall, but instead from the sum of the effective sample sizes of each study.^7^ Indeed, take the extreme example of meta-analyzing two studies, one with 1,000 cases and 0 controls and another one with 0 cases and 1,000 controls: the effective sample size of the meta-analysis is then 0, not 2,000. Misspecifying the GWAS sample size can lead to serious issues such as misestimating the SNP heritability.^7^ Fortunately, an overestimated sample size can be detected from the QC plot we propose, where the slope is then less than 1 for case-control studies using logistic regression. Here we have used this strategy to estimate reduced effective sample sizes in FinnGen GWAS summary statistics.

We have assessed the impact of these misspecifications in GWAS summary statistics on the predictive performance of some PGS methods. Using both the Bayesian LDpred2 models^15^ and our reimplementation of the frequentist lassosum model^16^ for deriving PGS, we have introduced and investigated some changes to possibly make these models more robust to misspecifications. Overall, these changes provided large improvements of predictive performance in the simulations with large misspecifications. The proposed QCs on GWAS summary statistics also provided better predictive performance for CAD, T1D, and vitamin D. However, these changes had limited effect when applied to some other real GWAS summary statistics, which is both unfortunate but also reassuring because it means that these GWAS summary statistics are of particularly good quality for follow-up analyses such as deriving PGS.

In conclusion, we recommend adopting these changes, i.e., performing the (refined) QC proposed here, forming independent LD blocks in the LD matrix, and using more regularization when needed. We also recommend using well-matched LD reference panels. More regularization can be achieved by testing additional smaller values for the heritability parameter in LDpred2-grid, and using the two new parameters introduced here in LDpred2-auto (section “LDpred2-low-h2 and LDpred2-auto-rob”). Note that LD blocks are already widely used by several methods, such as lassosum and PRS-CS, because they allow for processing smaller matrices at once.^16^^,^^19^ Imposing independent blocks on the LD matrix could result in further misspecifications,^14^ but here we have shown that well-defined blocks can actually make PGS methods more robust. PRS-CS is currently one of the most robust PGS methods; for example, it can use the (small) 1000G dataset as LD reference,^19^ and can even use a European LD reference panel with multi-ancestry GWAS summary statistics,^12^ also shown here. We believe this is made possible by the use of a strong regularization in PRS-CS ($\varphi^{-1}\psi_{j}^{-1}\geq 1$, which would approximately correspond to using s=0.5 in lassosum, δ=1 in lassosum2, and the new parameter shrink_corr=0.5 in LDpred2-auto). Using enough regularization is good for robustness, but note that using too much regularization can also damage predictive performance. To address this limitation, we recommend reliance on a proper QC and choice of ancestry-matched LD instead of on too much regularization. We would like to encourage large biobanks, such as FinnGen and Biobank Japan, to provide LD reference matrices matching the large GWAS summary statistics they provide, ideally based on the same large number of individuals. As a future research direction, we are interested in using multi-ancestry-matched LD matrices to use with multi-ancestry GWAS summary statistics to improve polygenic prediction in all ancestries. It would also be useful to investigate the impact of the QC, well-matched LD reference panels and other adjustments we propose here on other (non-PGS) methods, for which consistency and robustness are likely to be very important as well.^4^ For example, we are interested in assessing the impact of these misspecifications on the inference of disease architecture parameters in future work.

## Data Availability

The UK Biobank data is available through a procedure described at https://www.ukbiobank.ac.uk/using-the-resource/. All code used for this paper is available at https://github.com/privefl/paper-misspec/tree/master/code. We have extensively used R packages bigstatsr and bigsnpr^23^ for analyzing large genetic data, packages from the future framework^48^ for easy scheduling and parallelization of analyses on the HPC cluster, and packages from the tidyverse suite^49^ for shaping and visualizing results. The latest version of R package bigsnpr can be installed from GitHub, and a recent version can be installed from CRAN.

## Web resources

A tutorial on running LDpred2 and lassosum2 using R package bigsnpr is available at https://privefl.github.io/bigsnpr/articles/LDpred2.html.

We have recomputed the allele frequencies and imputation INFO scores within the European subset used here and across all imputed variants in the UK Biobank, and have made them available at https://doi.org/10.6084/m9.figshare.16635388.

We have formed independent LD blocks within the Northwestern European LD references provided in Privé et al.,^15^ and have made these updated versions available at https://doi.org/10.6084/m9.figshare.19213299.

## Supplemental information

Document S1. Figures S1–S39

Document S2. Article plus supplemental information
