# Supplementary material for: Identifying and correcting for misspecifications in GWAS summary statistics and polygenic scores
Source: HGG Adv. 2022 Aug 18;3(4):100136. doi: 10.1016/j.xhgg.2022.100136 (PMC9465343; doi:10.1016/j.xhgg.2022.100136)
Supplement: Document S1. Figures S1–S39 [file mmc1.pdf]

HGGA, Volume *3*

Supplemental information

Identifying and correcting for misspecifications in GWAS summary statistics and polygenic scores

Florian Privé, Julyan Arbel, Hugues Aschard, and Bjarni J. Vilhjálmsson

Supplementary Materials

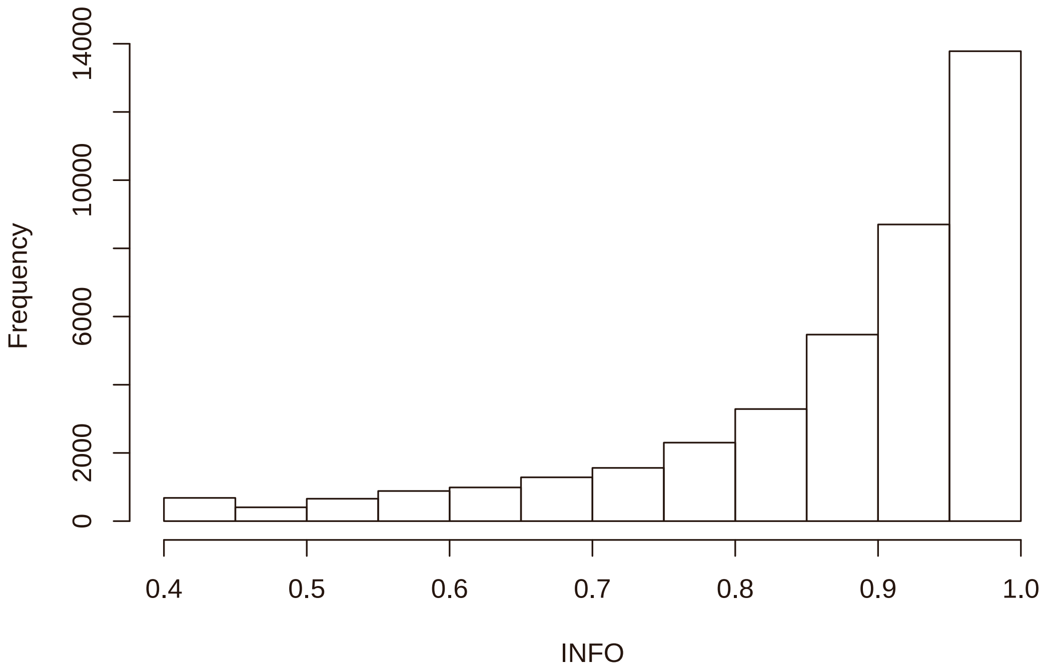

Figure S1: Histogram of the imputation INFO scores of the 40,000 variants from chromosome 22 of the UK Biobank data used in the simulations.

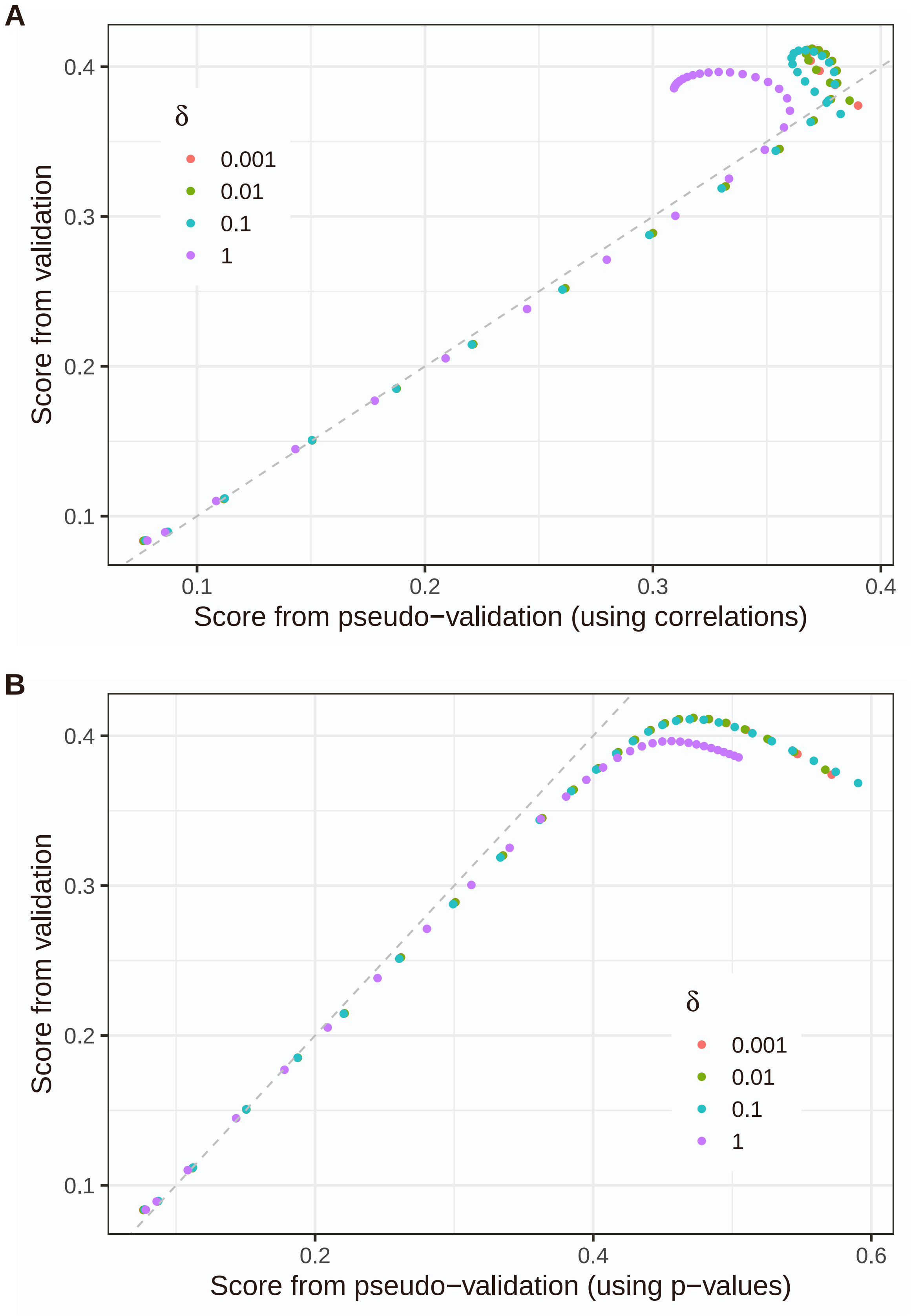

Figure S2: For one simulation, scores from validation versus scores from pseudo-validation as described in Mak et al.[1] using either **A:** correlations (the default in lassosum) or **B:** p-values, when computing local false discovery rates.

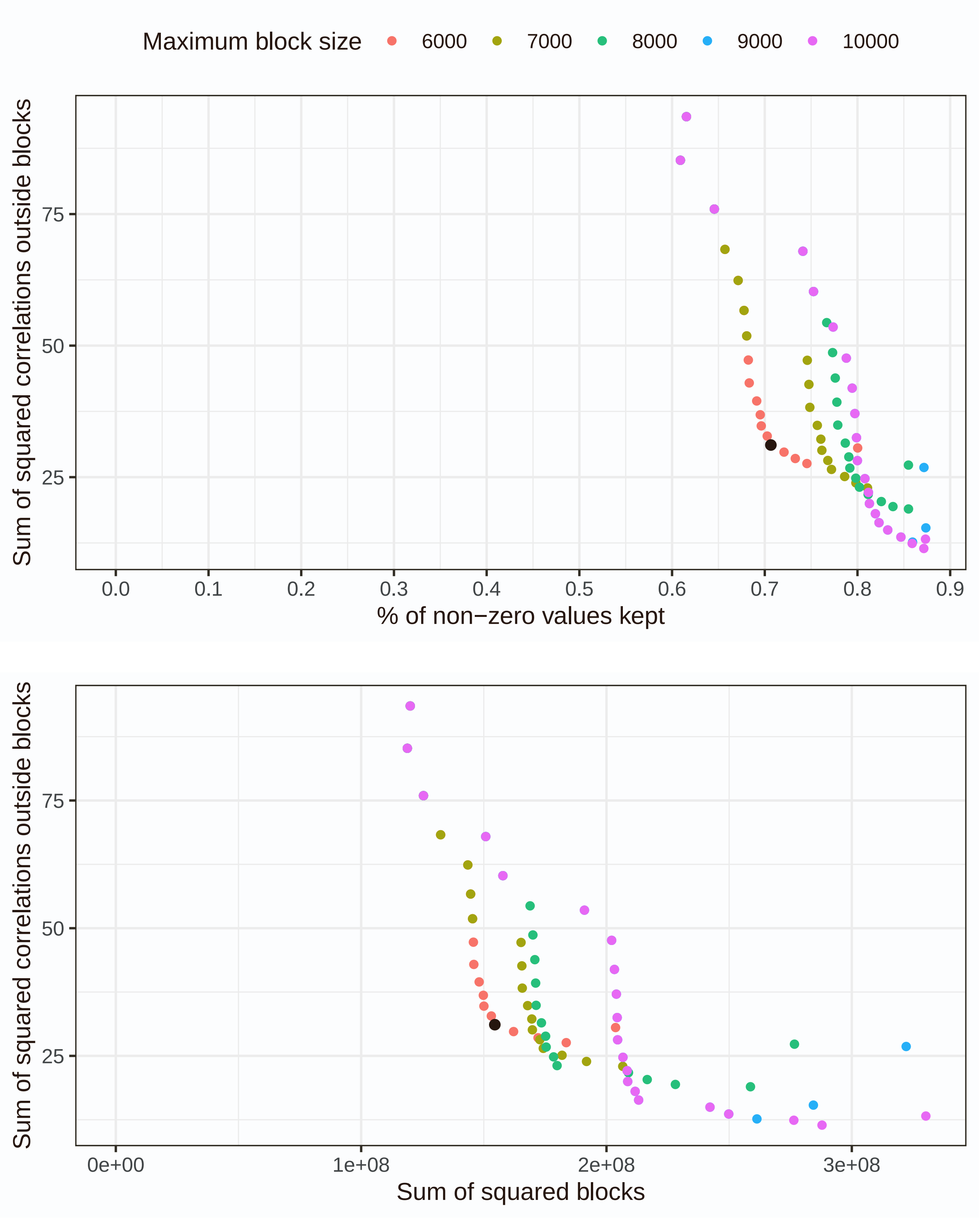

Figure S3: Results of different block splits of the LD matrix of chromosome 22 used in simulations, for different numbers of blocks and maximum number of variants in each block. The black point corresponds to the final split chosen.

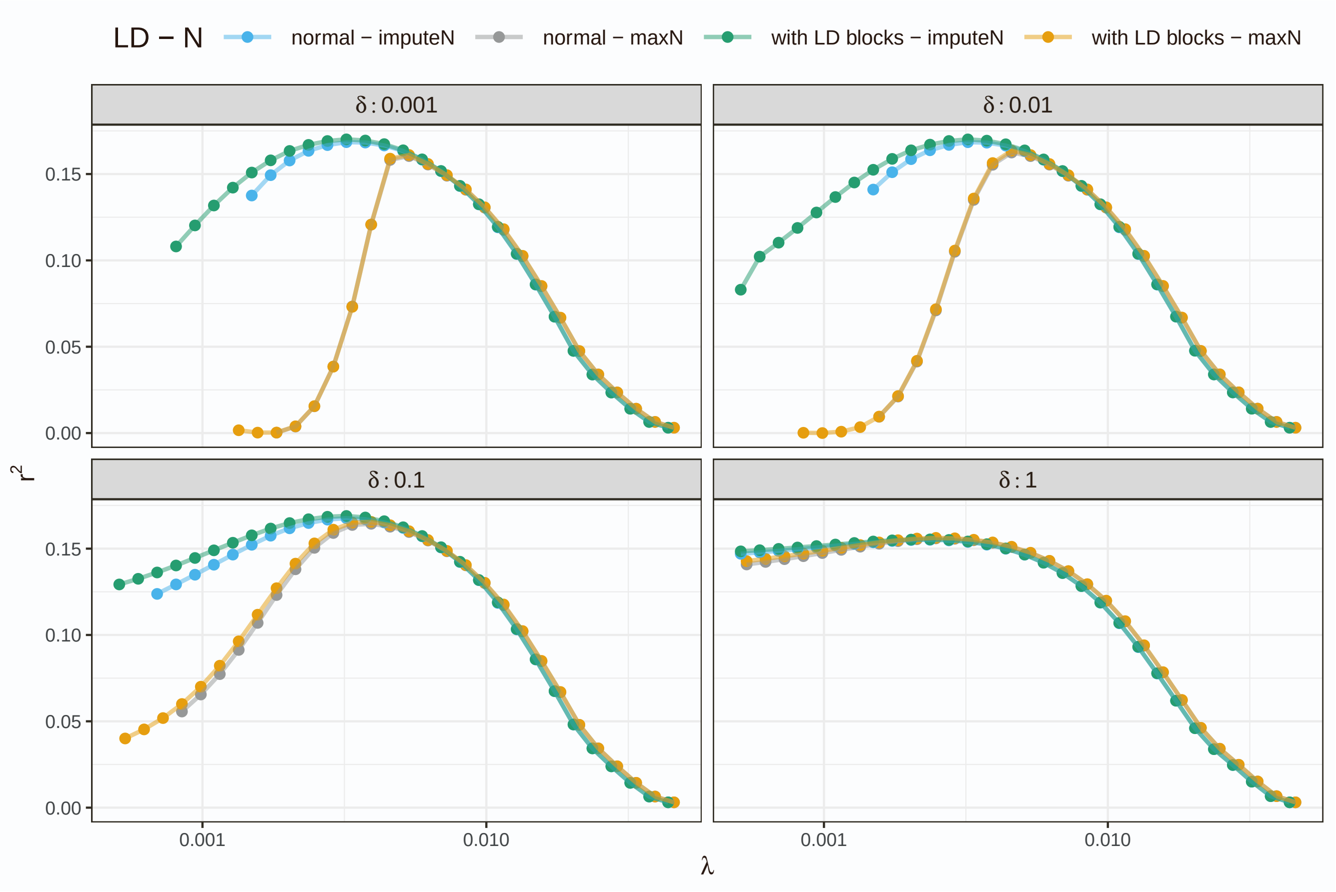

Figure S4: Multiple results (squared correlation r^2 between polygenic score and phenotype) over a grid of parameters (λ and δ) for lassosum2 in one of the simulations with misspecified GWAS sample sizes (n_j). The different colors indicate whether we use the maximum of n_j's ("maxN") or the imputed n_j's ("imputeN"), and the normal LD matrix ("normal") or the one with independent LD blocks ("with LD blocks"). Missing points represent models that were detected as divergent.

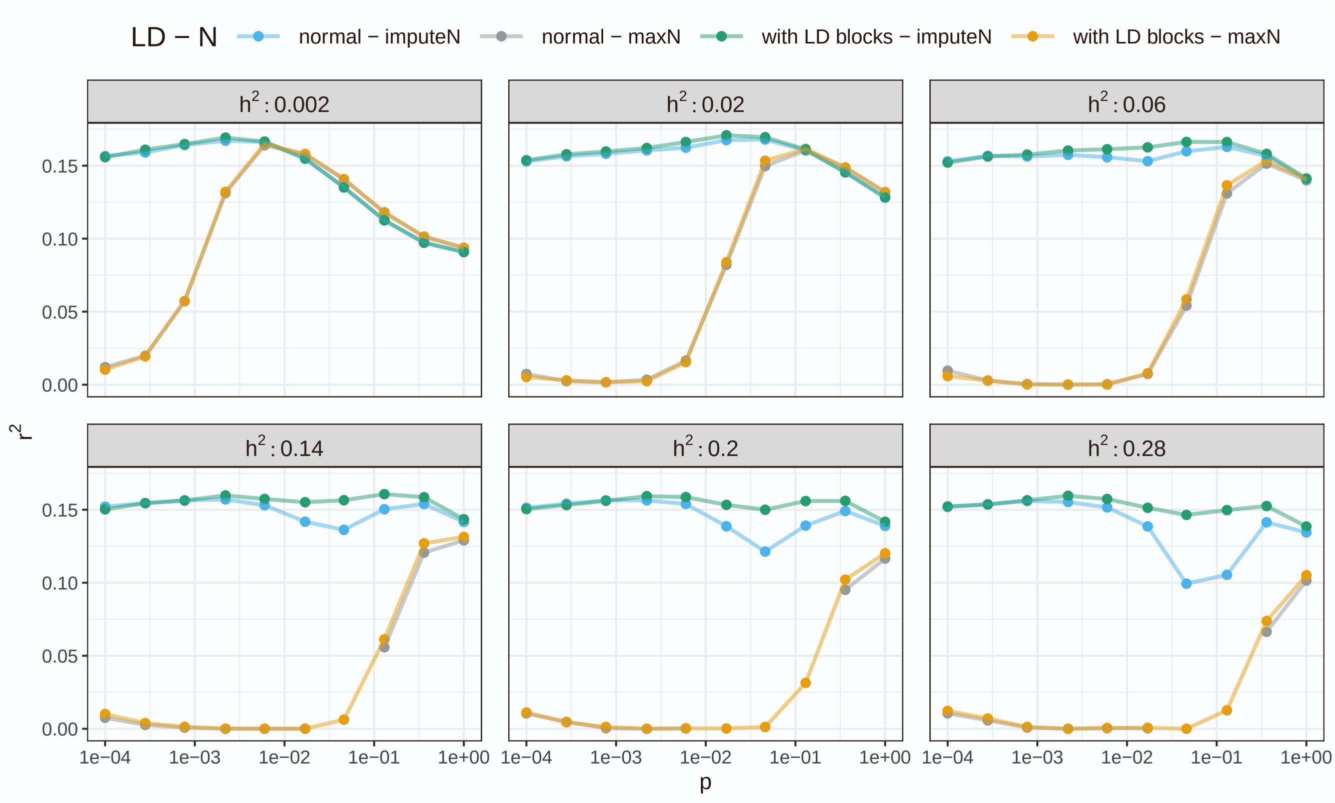

Figure S5: Multiple results (squared correlation r^2 between polygenic score and phenotype) over a grid of parameters (the SNP heritability h^2 and the proportion of causal variants p) for LDpred2(-grid) in one of the simulations with misspecified GWAS sample sizes (n_j). The different colors indicate whether we use the maximum of n_j's ("maxN") or the imputed n_j's ("imputeN"), and the normal LD matrix ("normal") or the one with independent LD blocks ("with LD blocks"). Note that the small h^2 values (0.002, 0.02, 0.06) were added and are part of the method we call "LDpred2-low-h2" in the main text. Missing points represent models that were detected as divergent.

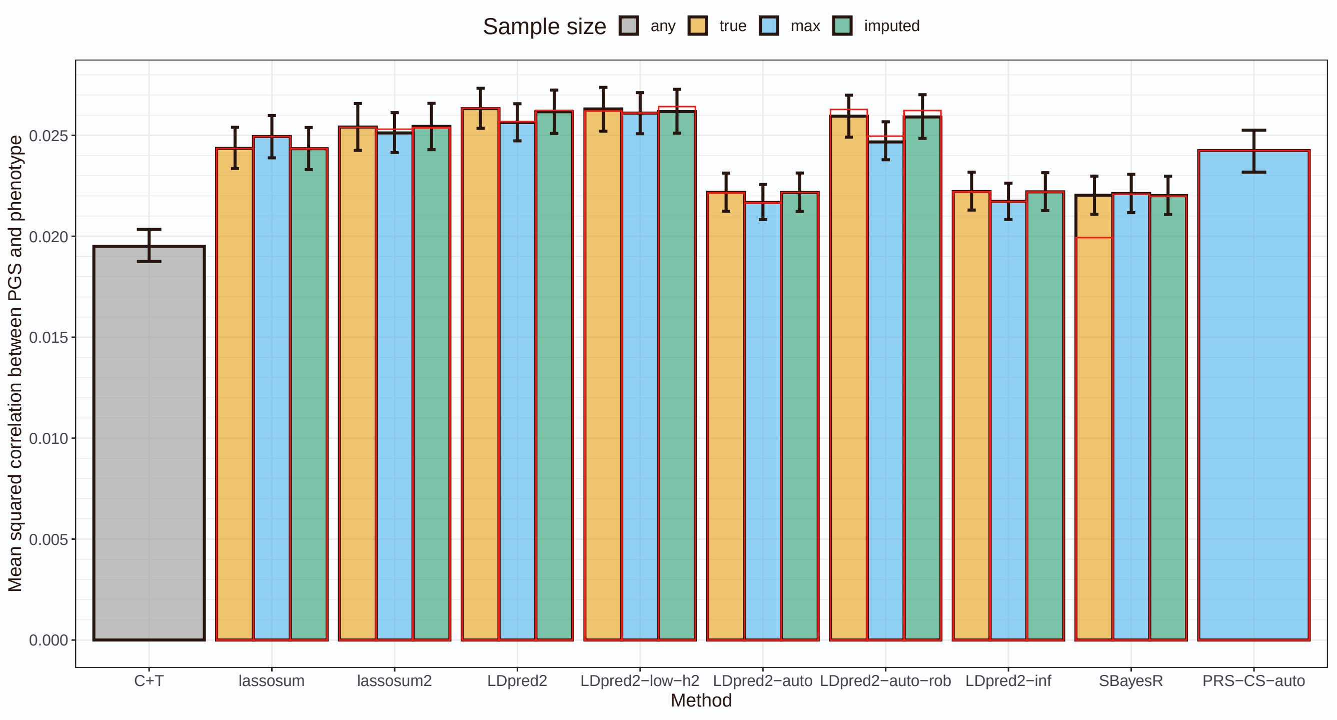

Figure S6: Results for the simulations with sample size misspecification and a heritability of 4%, averaged over 10 simulations for each scenario. Reported 95% confidence intervals are computed from 10,000 non-parametric bootstrap replicates of the mean. The GWAS sample size is either “true” when providing the true per-variant sample size, “max” when providing instead the maximum sample size as a unique value to be used for all variants, “imputed”, or “any” when the method does not use this information (the case for C+T). Red bars correspond to using the LD with independent blocks, which is a requirement in lassosum and PRS-CS.

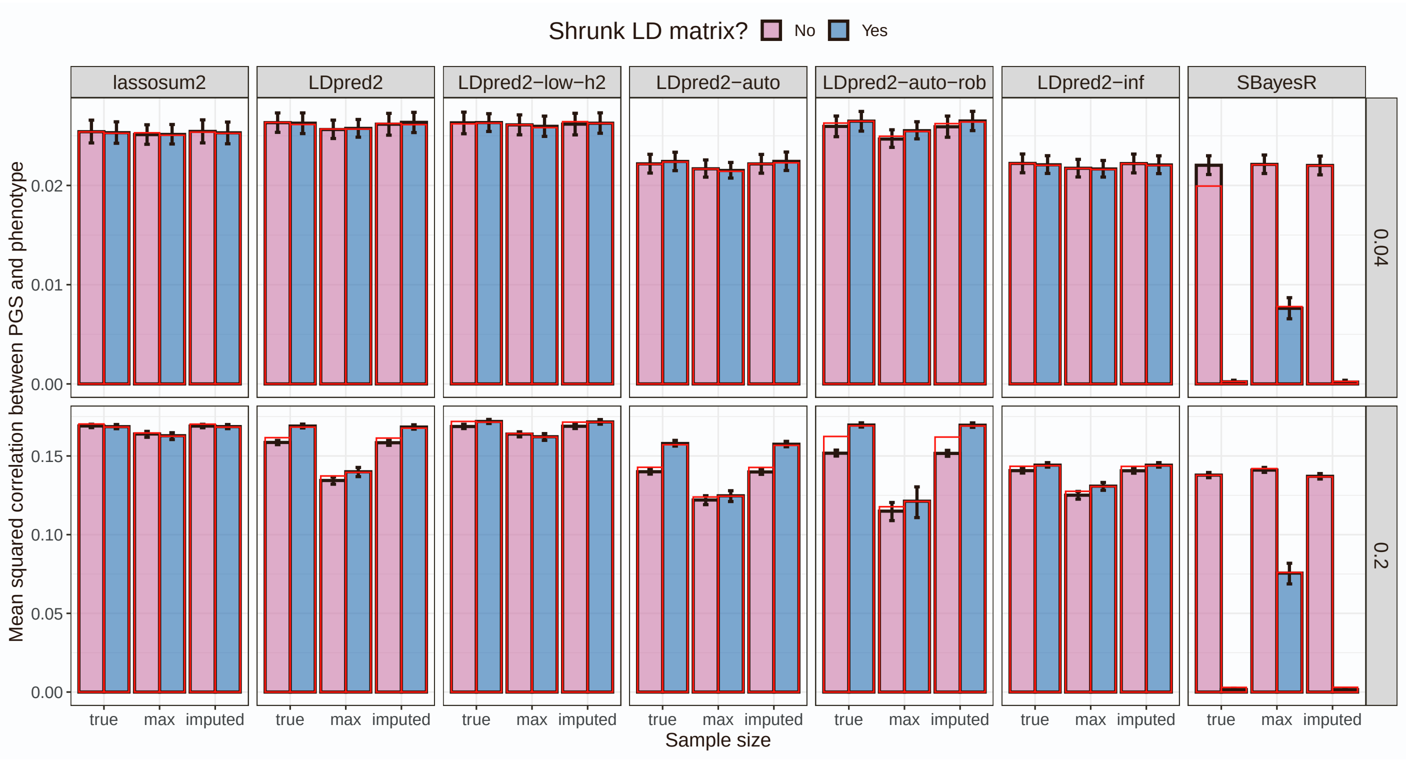

Figure S7: Results for the simulations with sample size misspecification and a heritability of 20% (bottom panels) or 4% (top panels), averaged over 10 simulations for each scenario. Reported 95% confidence intervals are computed from 10,000 non-parametric bootstrap replicates of the mean. The GWAS sample size is either "true" when providing the true per-variant sample size, "max" when providing instead the maximum sample size as a unique value to be used for all variants, or "imputed". Red bars correspond to using the LD with independent blocks. Bars filled in blue correspond to using the shrunk LD matrix computed from GCTB[2], otherwise the windowed LD matrix from LDpred2 is used.

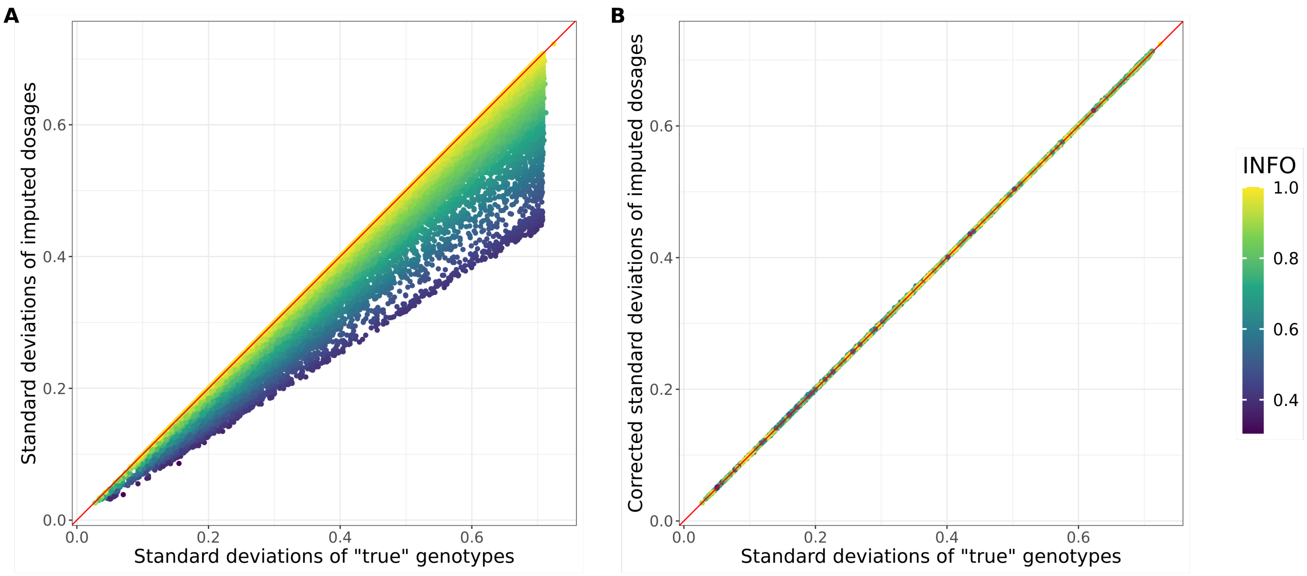

Figure S8: **A:** Raw standard deviations (SDs) from imputed dosages or **B:** corrected SDs from imputed dosages (dividing them by $\sqrt{\text{INFO}}$) versus SDs from "true" genotype calls, colored by INFO scores.

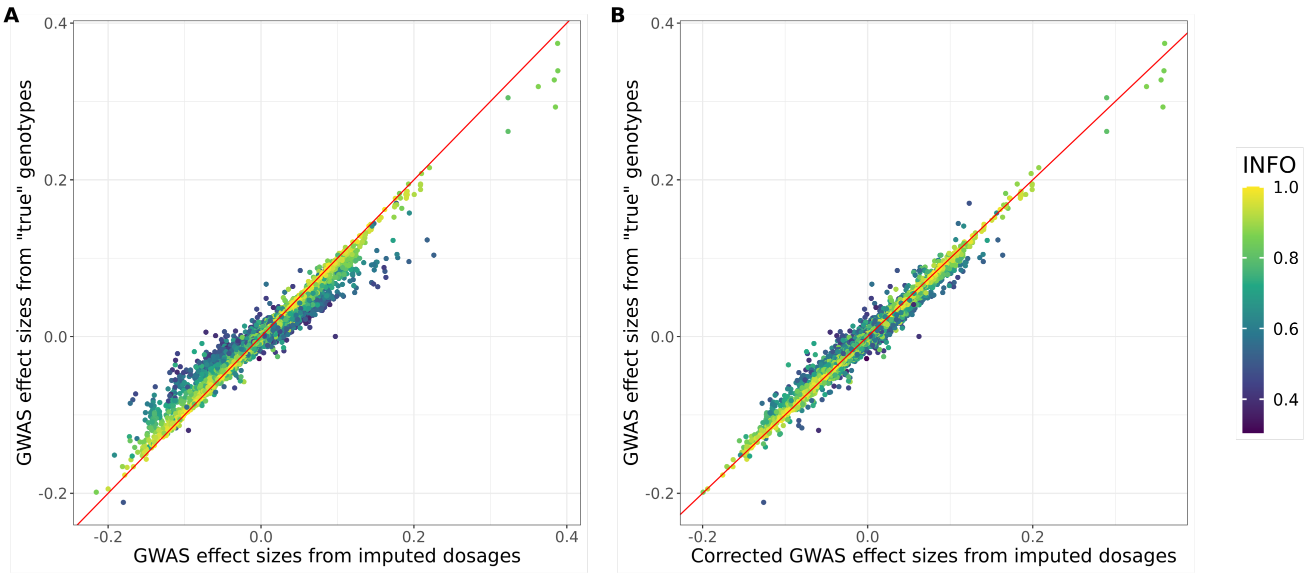

Figure S9: GWAS effect sizes ($\hat{\gamma}$) from "true" genotype calls versus either **A:** raw $\hat{\gamma}$ from imputed dosages or **B:** corrected $\hat{\gamma}$ from imputed dosages (multiplying them by $\sqrt{\text{INFO}}$), colored by INFO scores.

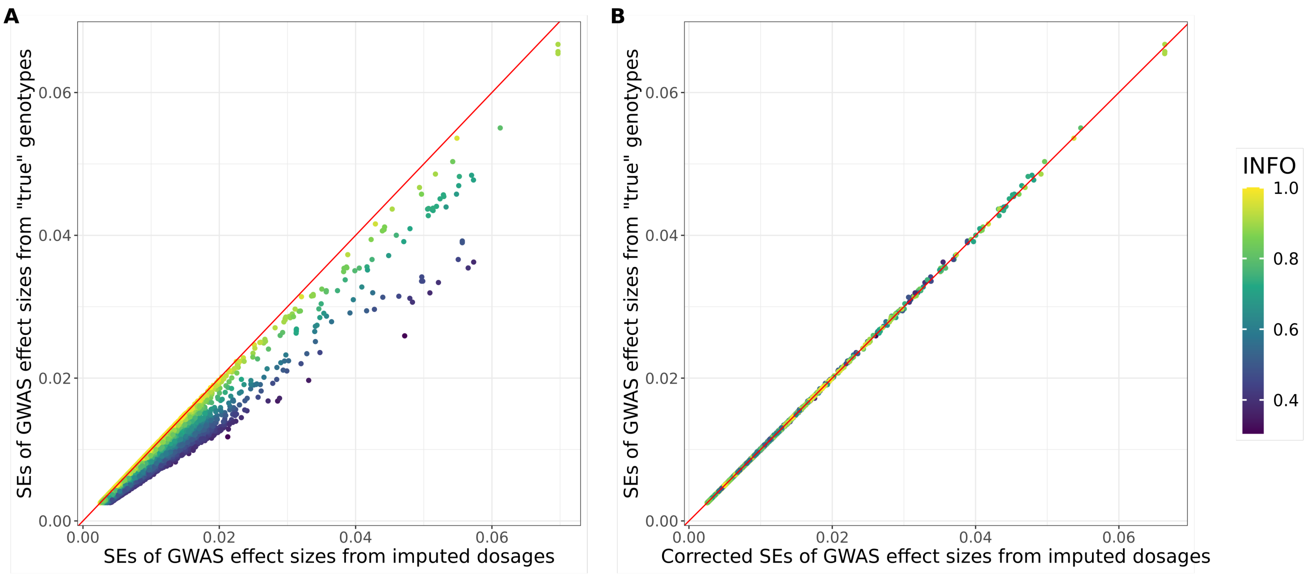

Figure S10: Standard errors of GWAS effect sizes (SEs) from "true" genotype calls versus either **A:** raw SEs from imputed dosages or **B:** corrected SEs from imputed dosages (multiplying them by $\sqrt{\text{INFO}}$), colored by INFO scores.

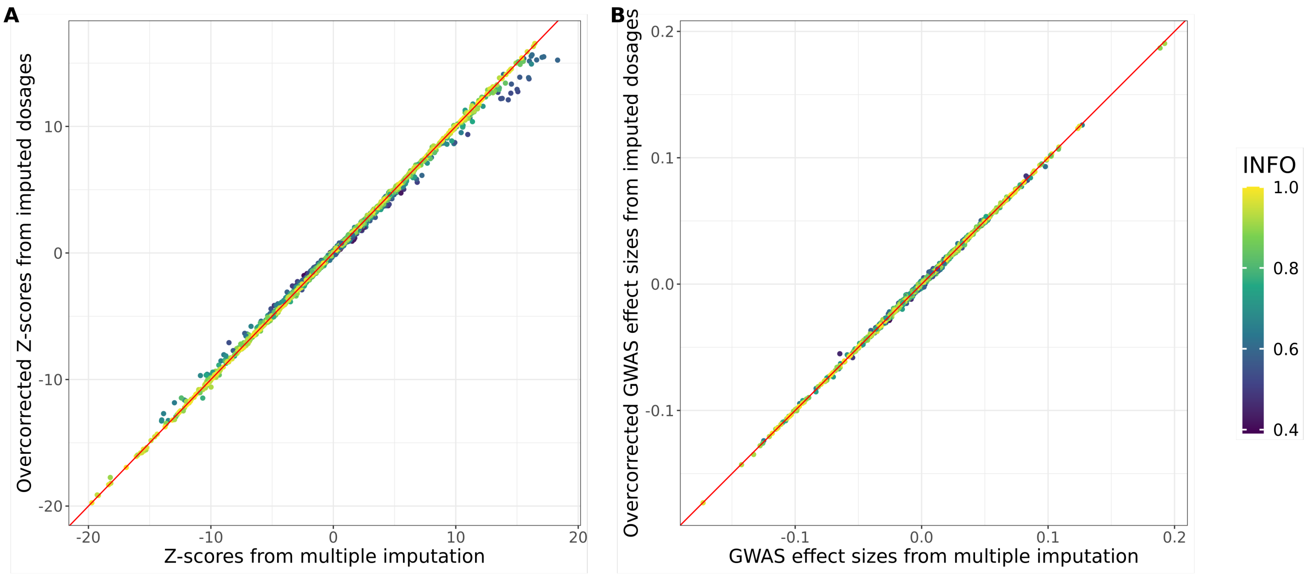

Figure S11: (Over)corrected GWAS **A:** Z-scores and **B:** effect sizes from imputed dosages (multiplying them by INFO) versus the ones obtained from multiple imputation (MI, 20 draws used, Palmer and Pe'er[3]), colored by INFO scores. This shows only one tenth of the simulated variants, because MI can be computationally intensive.

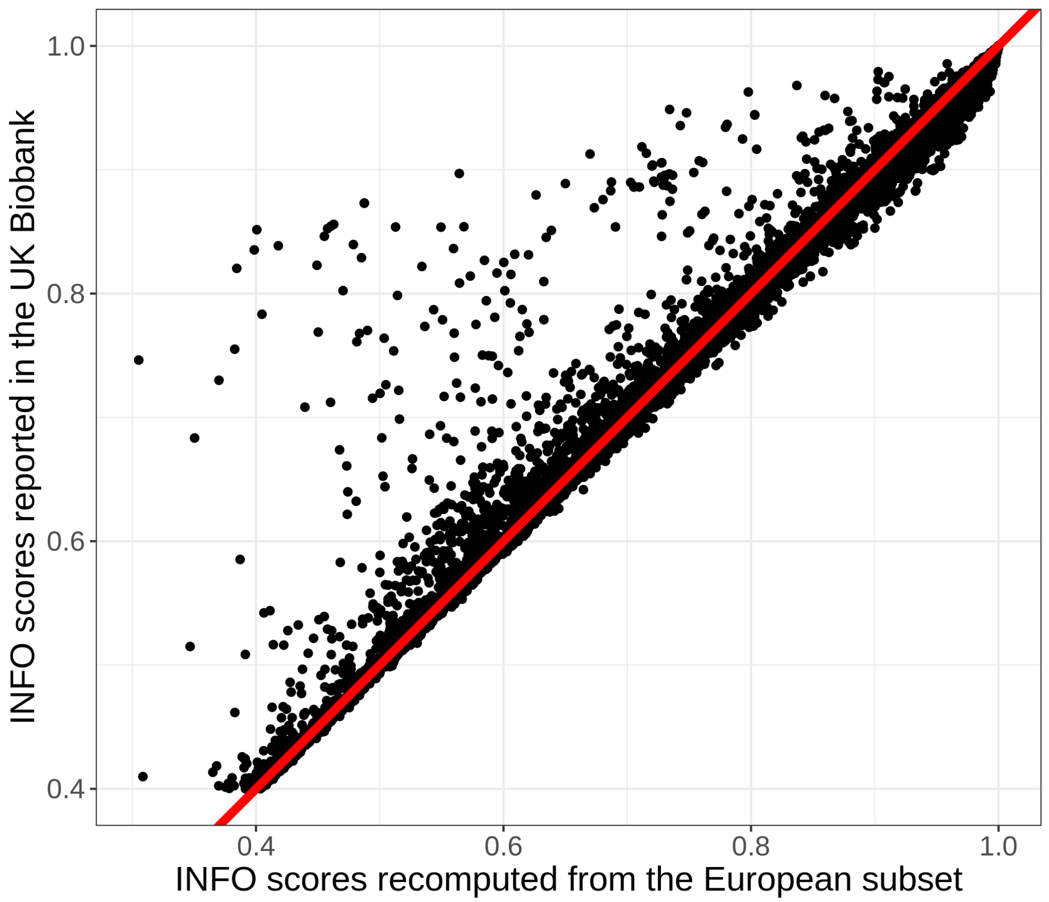

Figure S12: INFO scores reported in the UK Biobank for variants used in simulations versus INFO scores recomputed from the subset of 362,307 European individuals used in this paper.

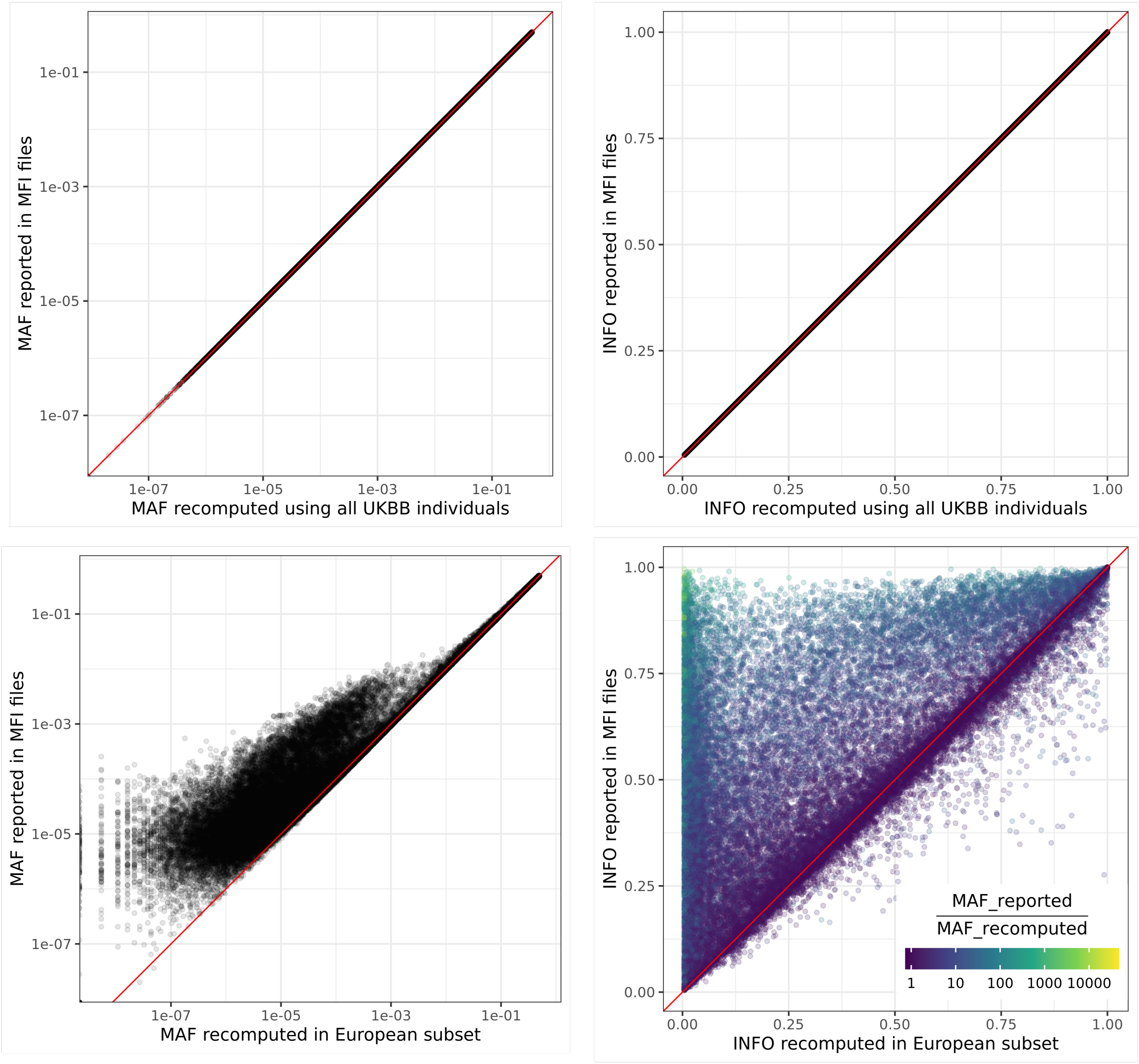

Figure S13: Minor allele frequencies (MAF) and INFO scores in the UK Biobank for 50,000 variants on chromosome 22 (chosen at random). These are either reported (in MFI files from the UK Biobank), recomputed from the whole data or from the subset of 362,307 European individuals used in this paper. MAF are represented on a log scale.

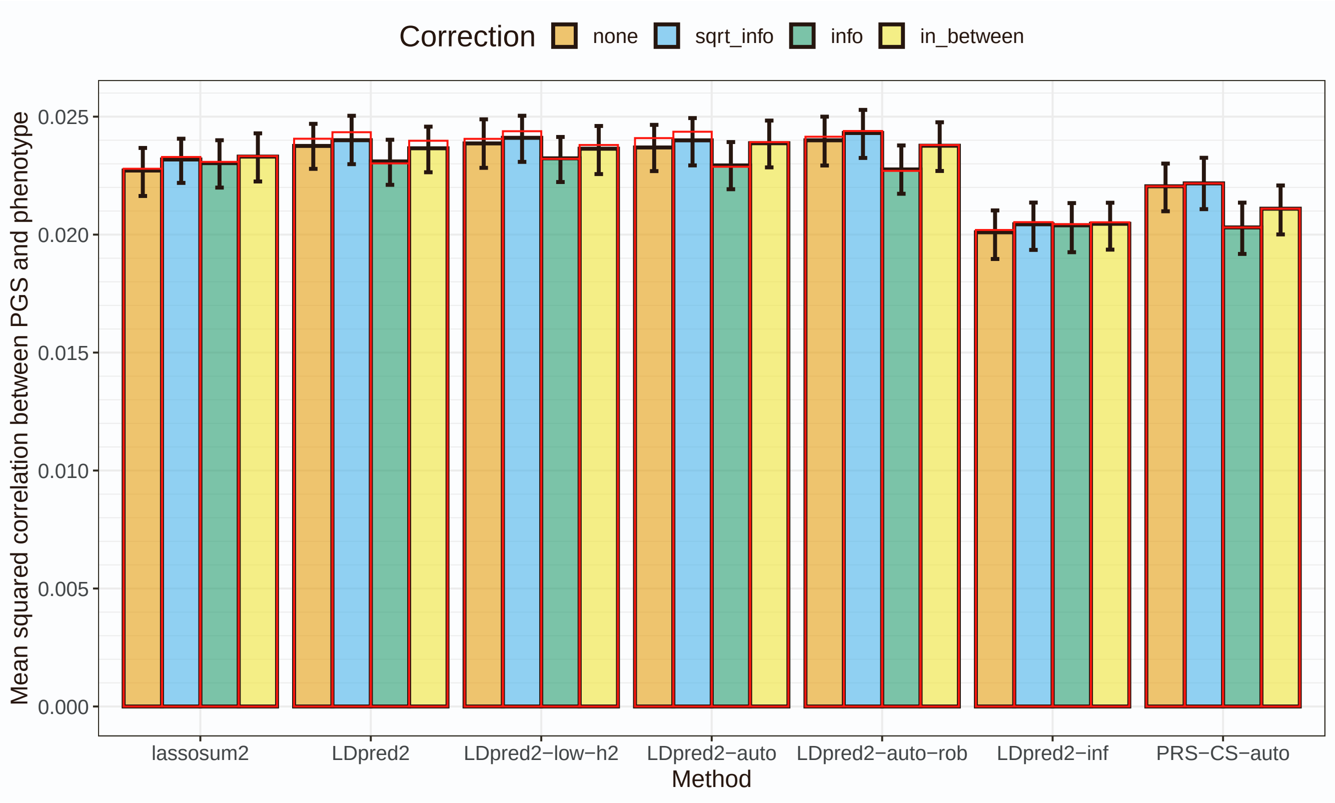

Figure S14: Results of predictive performance for the simulations (with a heritability of 4%) using GWAS summary statistics from imputed dosage data, averaged over 10 simulations for each scenario. Reported 95% confidence intervals are computed from 10,000 non-parametric bootstrap replicates of the mean. Correction "sqrt_info" corresponds to using $\hat{\gamma}_j^{\text{imp}} \cdot \sqrt{\text{INFO}_j}$ and $\text{se}(\hat{\gamma}_j)^{\text{imp}} \cdot \sqrt{\text{INFO}_j}$. Correction "info" corresponds to using $\hat{\gamma}_j^{\text{imp}} \cdot \text{INFO}_j$ and $n_j \cdot \text{INFO}_j$. Correction "in_between" corresponds to using $\hat{\gamma}_j^{\text{imp}} \cdot \text{INFO}_j$, $\text{se}(\hat{\gamma}_j)^{\text{imp}} \cdot \sqrt{\text{INFO}_j}$, and $n_j \cdot \text{INFO}_j$. Red bars correspond to using the LD with independent blocks.

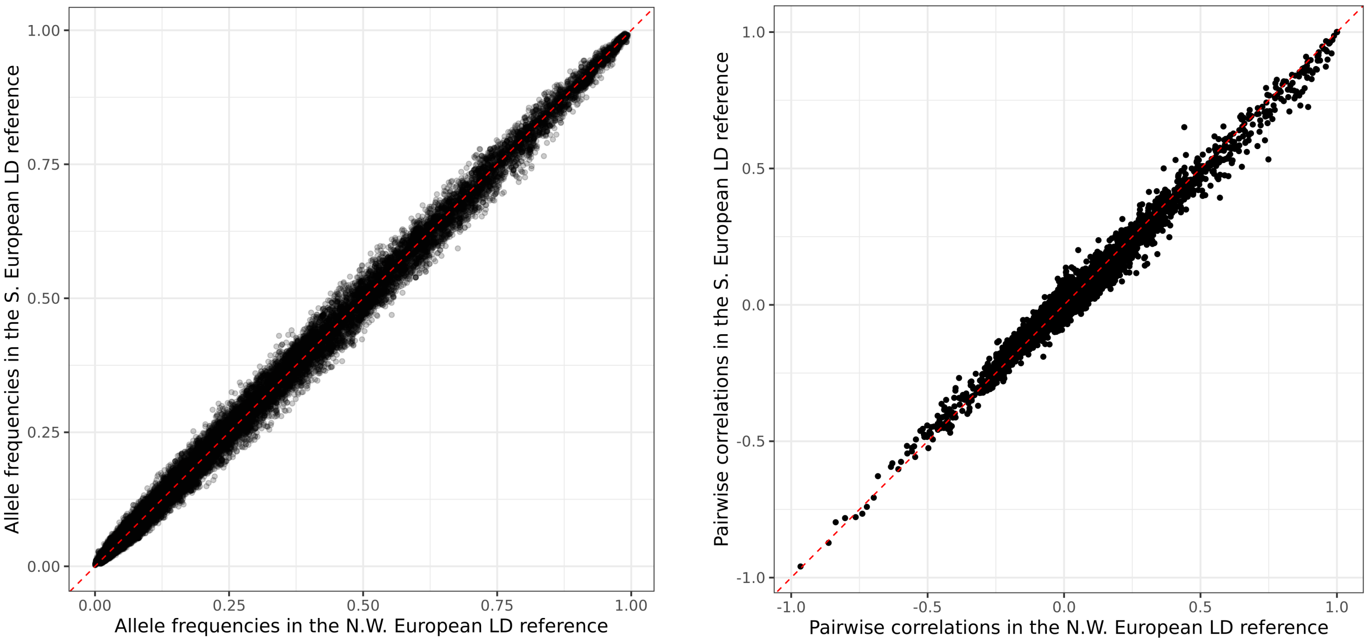

Figure S15: Comparison of allele frequencies and pairwise correlations between the two LD reference panels used in the simulations, one from North-West Europe and the alternative one from South Europe.

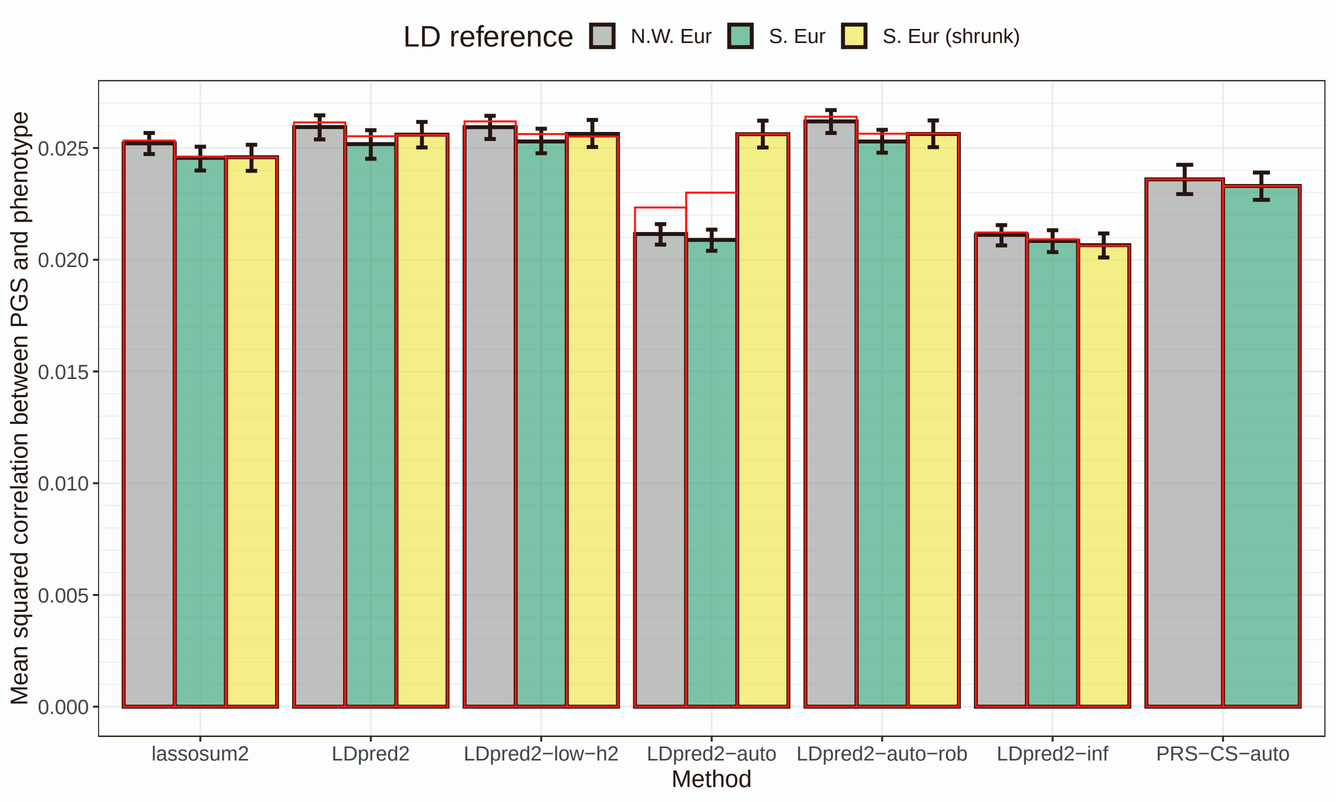

Figure S16: Results for the simulations with summary statistics with LD matrices based on two different populations, using a heritability of 4% (instead of 20%). One comes from the same ancestry used for computing the GWAS summary statistics (North-West Europe), while the other one comes from South Europe (alternative LD reference). Reported 95% confidence intervals are computed from 10,000 non-parametric bootstrap replicates of the mean. Red bars correspond to using the LD with independent blocks (Methods), which is a requirement for PRS-CS.

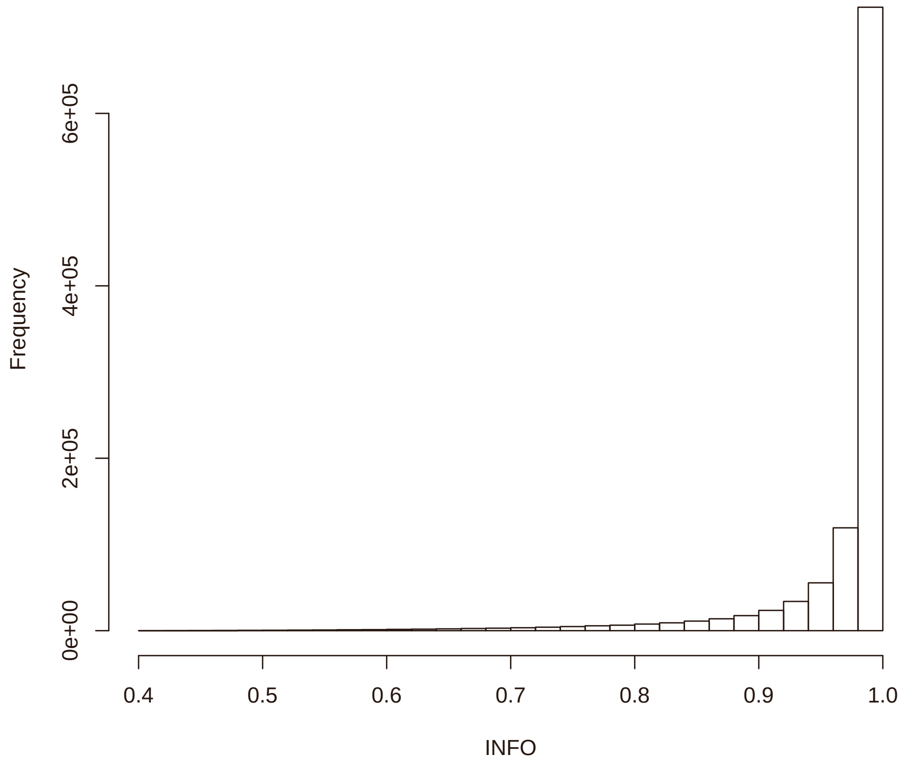

Figure S17: Histogram of the imputation INFO scores of the HapMap3 variants from the OncoArray GWAS summary statistics for breast cancer[4].

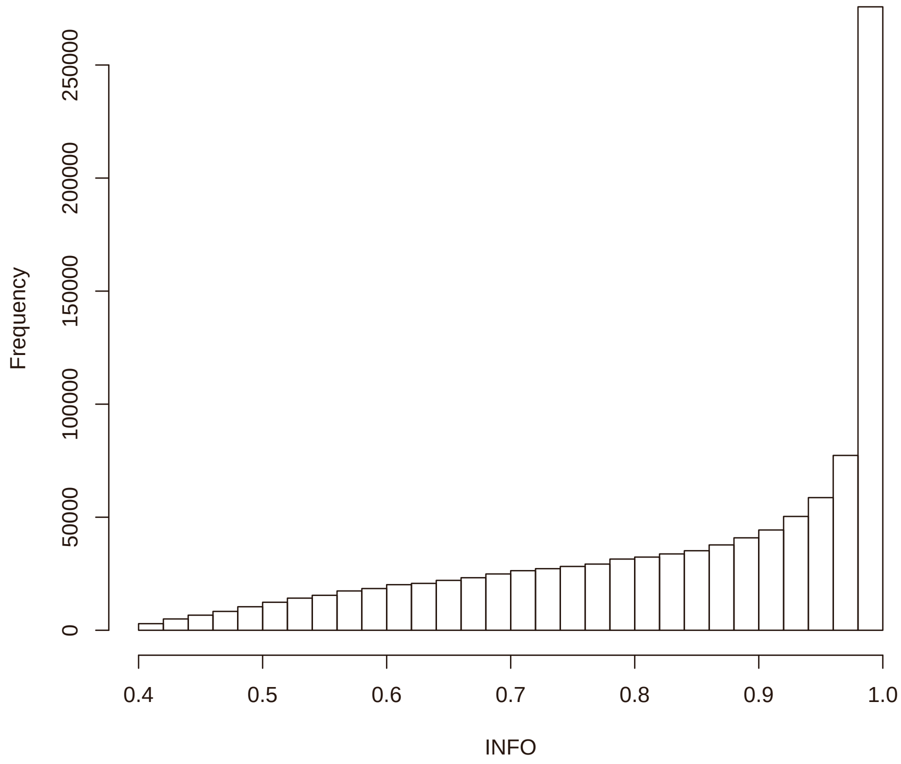

Figure S18: Histogram of the imputation INFO scores of the HapMap3 variants from the iCOGS GWAS summary statistics for breast cancer[5].

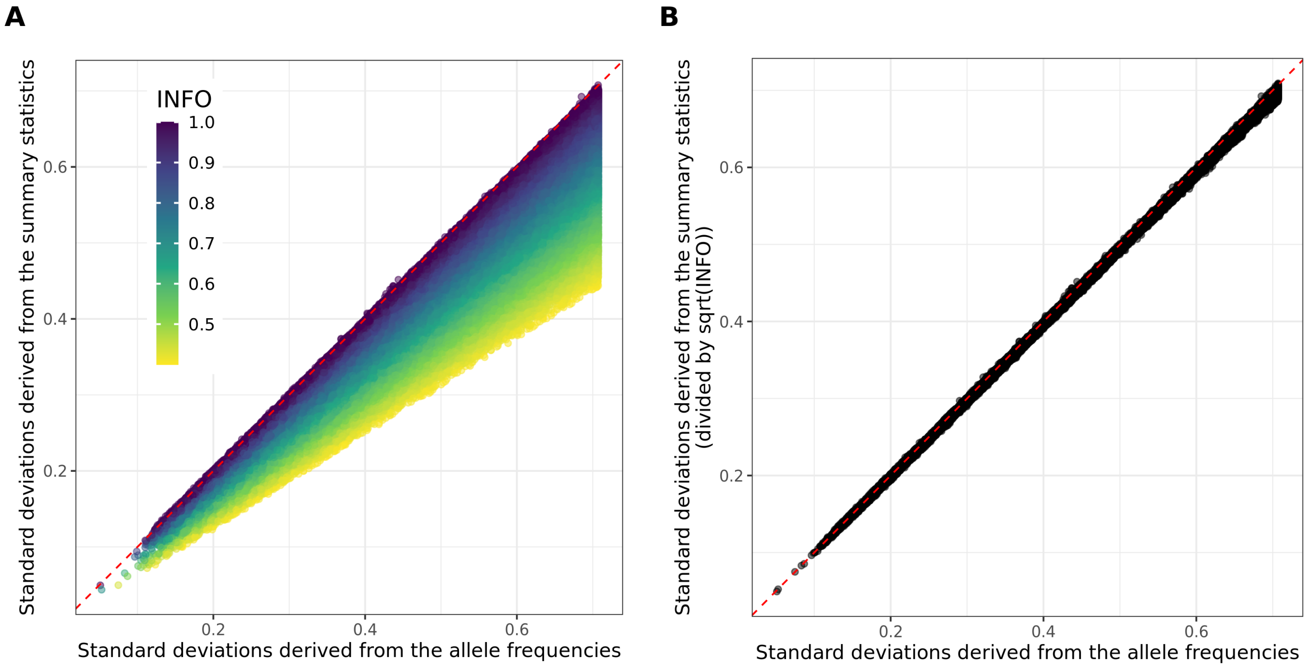

Figure S19: Standard deviations inferred from the iCOGS breast cancer GWAS summary statistics (**A:** Raw or **B:** dividing them by $\sqrt{\text{INFO}}$) versus the ones inferred from the reported GWAS allele frequencies. Only 100,000 variants are represented, at random.

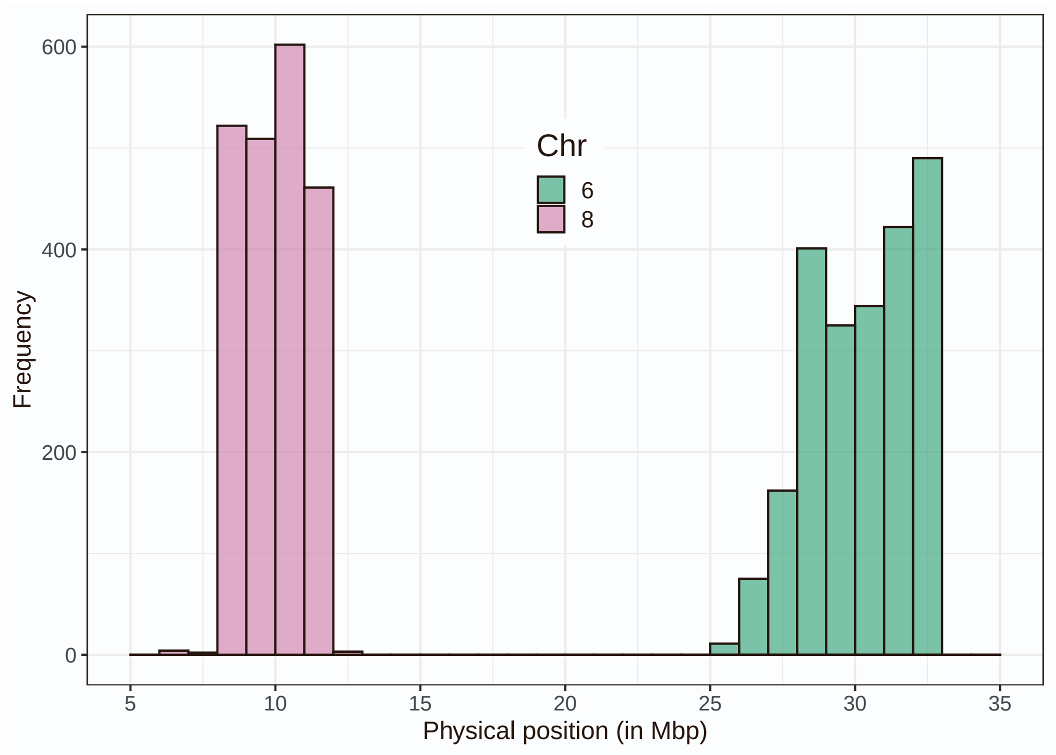

Figure S20: Histogram of the positions of outlier variants from Figure 4B in the main text.

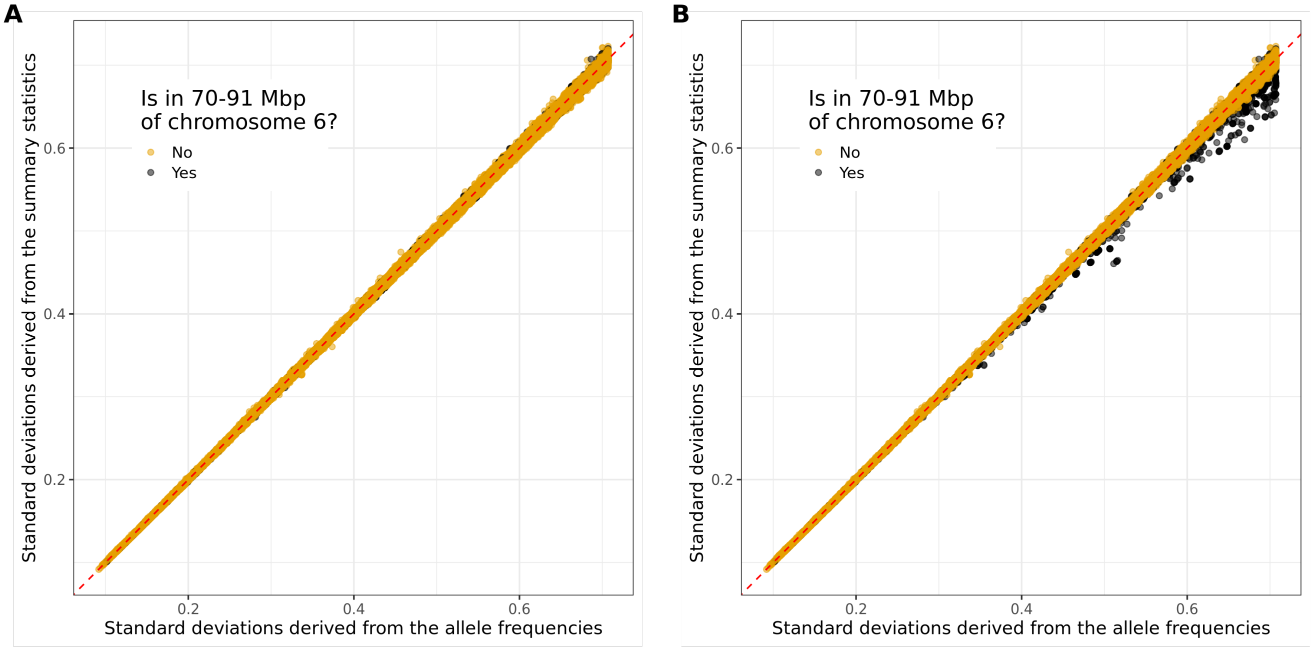

Figure S21: Standard deviations inferred from the simulated GWAS summary statistics (**A:** with no covariate; **B:** with PC19 from the UK Biobank as covariate) versus the ones inferred from the allele frequencies. Only HapMap3 variants from chromosome 6 are represented.

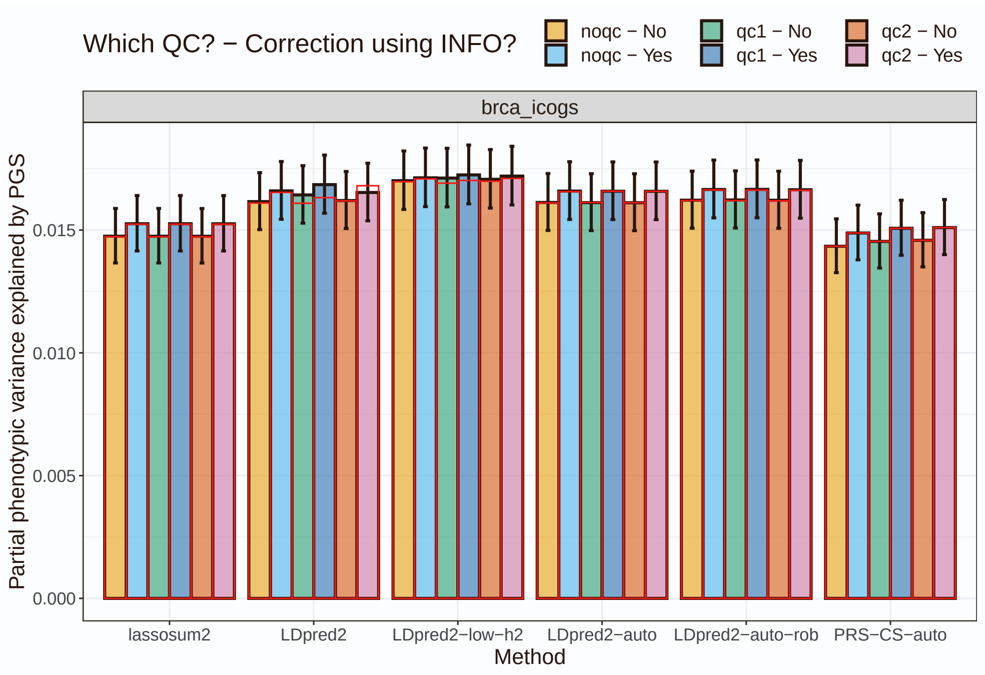

Figure S22: Variance explained of BRCA in the UK Biobank by PGS derived from external summary statistics (iCOGS). These are computed using function `pcor` of R package bigstatsr where 95% confidence intervals are obtained through Fisher's Z-transformation; these values are then squared.

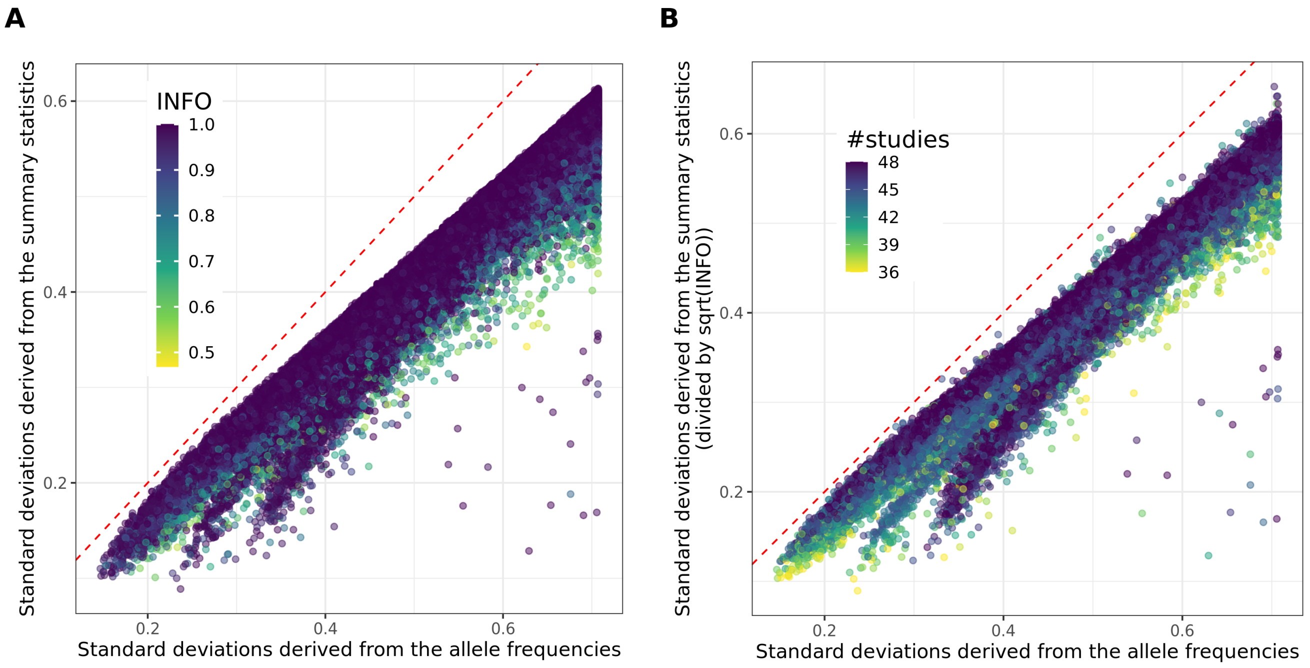

Figure S23: Standard deviations inferred from the CAD GWAS summary statistics (**A:** Raw or **B:** dividing them by $\sqrt{\text{INFO}}$) versus the ones inferred from the reported GWAS allele frequencies. Only 100,000 variants are represented, at random.

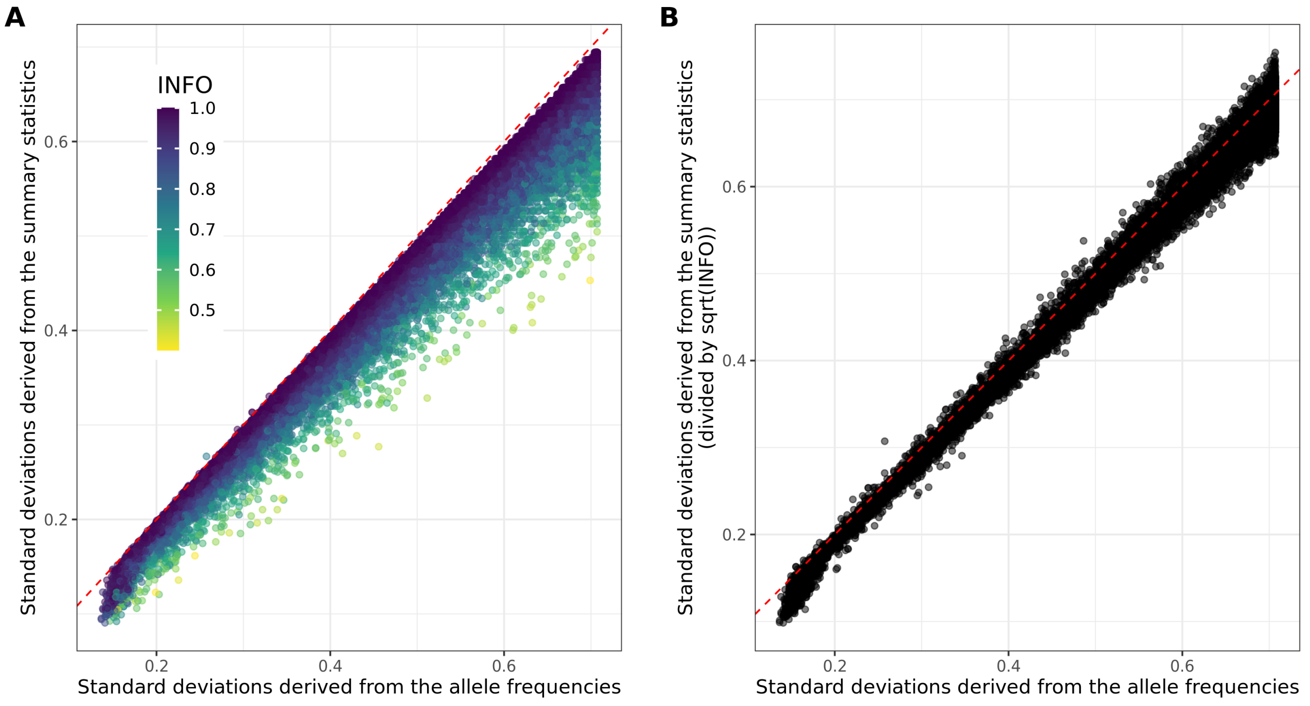

Figure S24: Standard deviations inferred from the MDD GWAS summary statistics (**A:** Raw or **B:** dividing them by $\sqrt{\text{INFO}}$) versus the ones inferred from the reported GWAS allele frequencies. Only 100,000 variants are represented, at random.

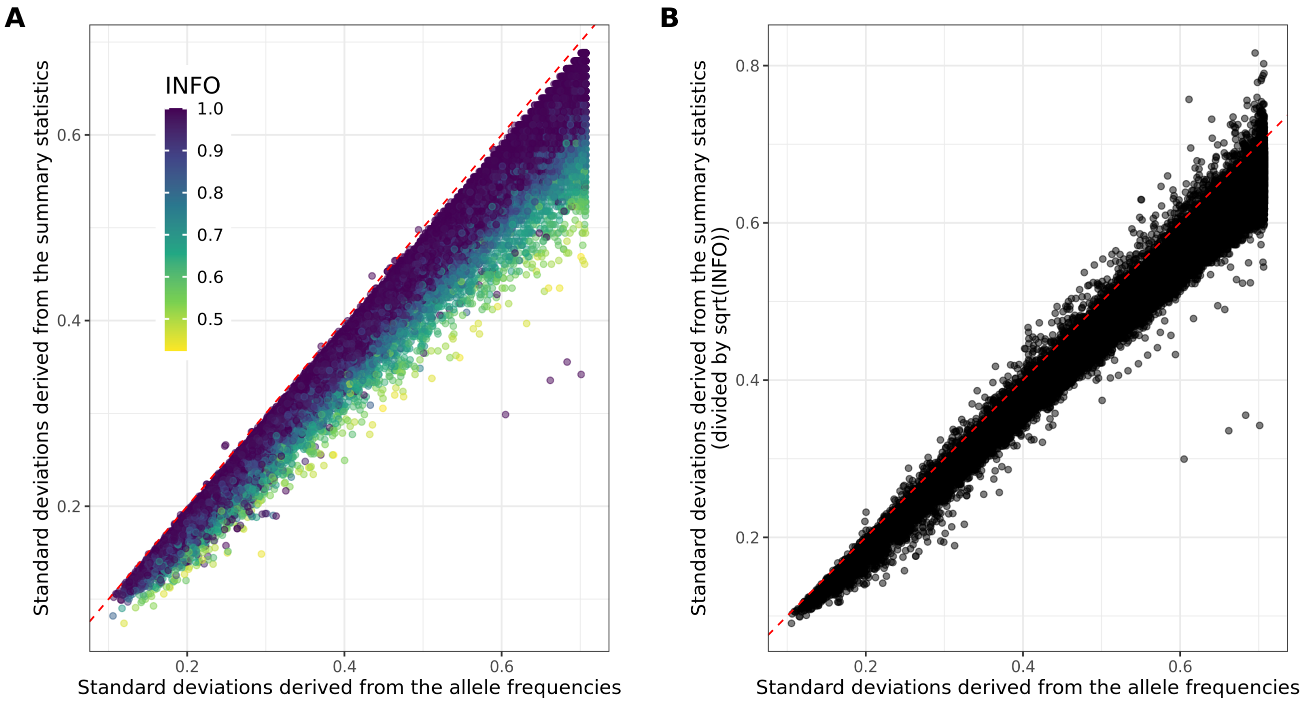

Figure S25: Standard deviations inferred from the PRCA GWAS summary statistics (**A:** Raw or **B:** dividing them by $\sqrt{\text{INFO}}$) versus the ones inferred from the reported GWAS allele frequencies. Only 100,000 variants are represented, at random.

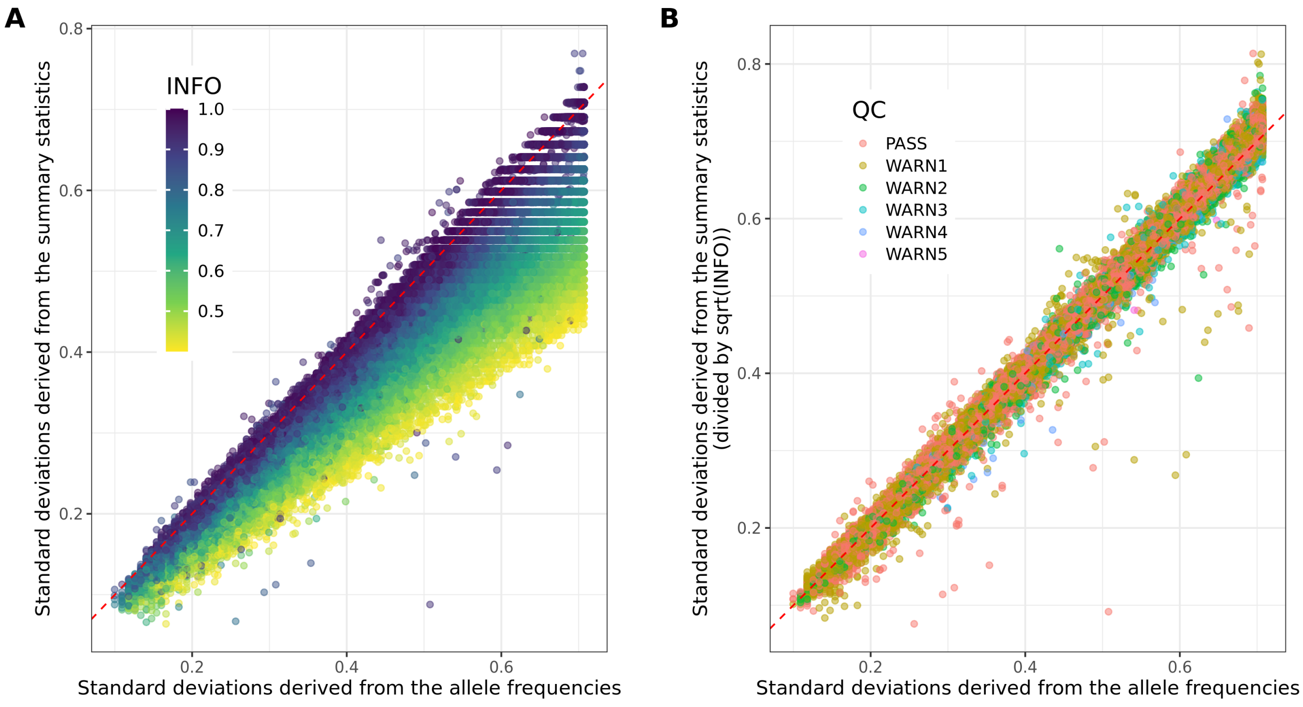

Figure S26: Standard deviations inferred from the T1D (Affymetrix) GWAS summary statistics (**A:** Raw or **B:** dividing them by $\sqrt{\text{INFO}}$) versus the ones inferred from the reported GWAS allele frequencies. Only 100,000 variants are represented, at random.

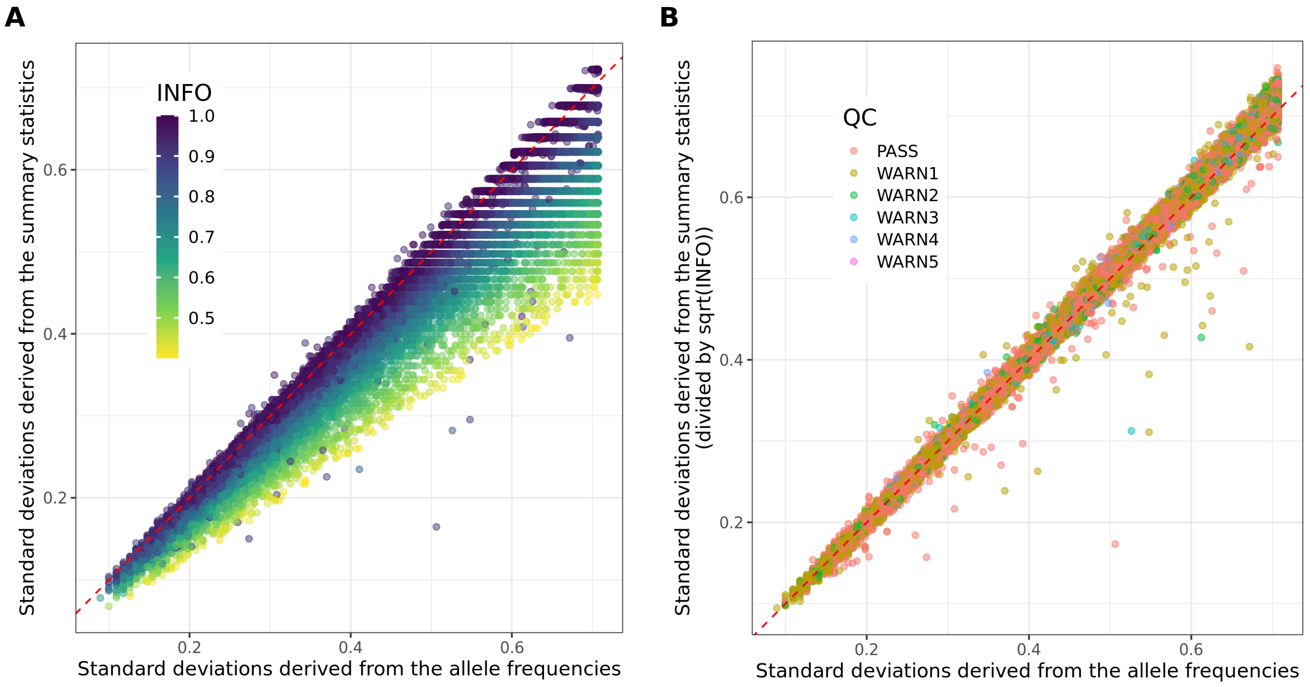

Figure S27: Standard deviations inferred from the T1D (Illumina) GWAS summary statistics (**A:** Raw or **B:** dividing them by $\sqrt{\text{INFO}}$) versus the ones inferred from the reported GWAS allele frequencies. Only 100,000 variants are represented, at random.

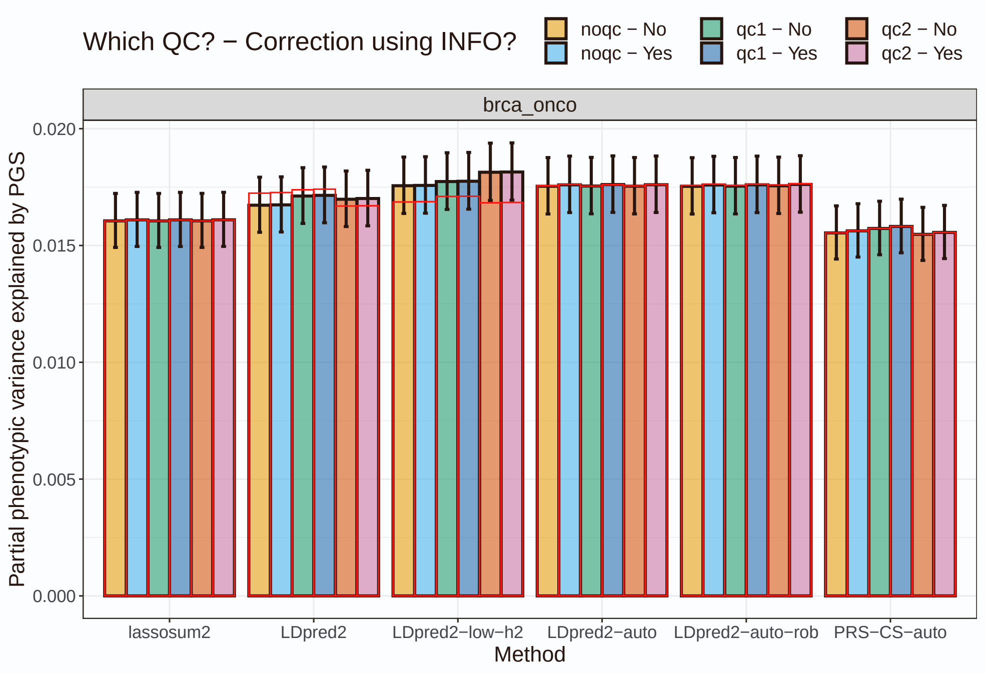

Figure S28: Variance explained of BRCA in the UK Biobank by PGS derived from external summary statistics (OncoArray). These are computed using function `pcor` of R package bigstatsr where 95% confidence intervals are obtained through Fisher's Z-transformation; these values are then squared.

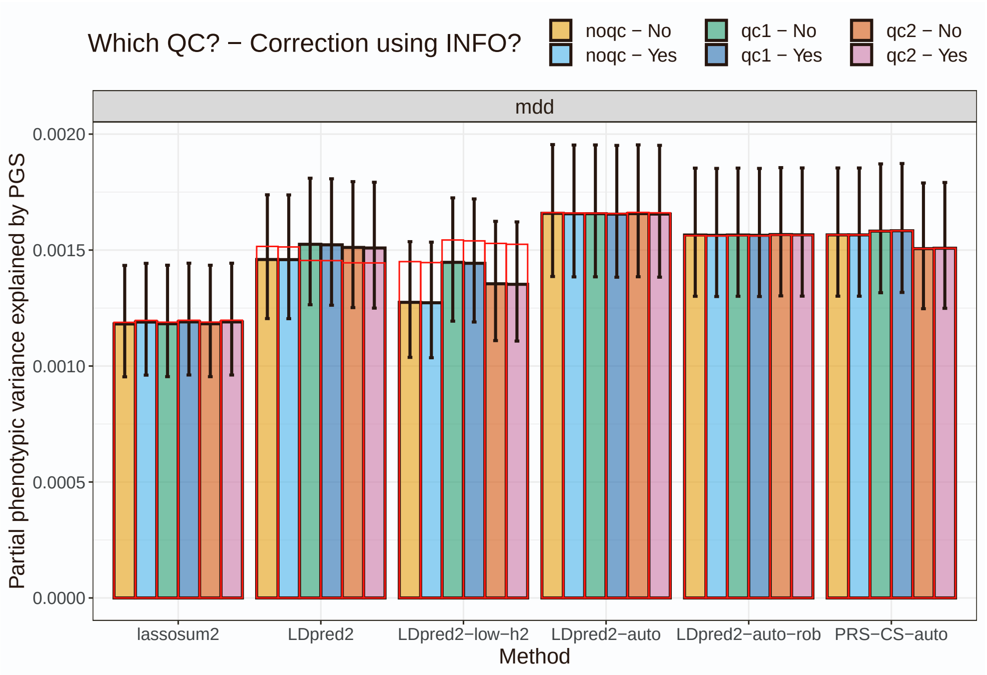

Figure S29: Variance explained of MDD in the UK Biobank by PGS derived from external summary statistics. These are computed using function `pcor` of R package bigstatsr where 95% confidence intervals are obtained through Fisher's Z-transformation; these values are then squared.

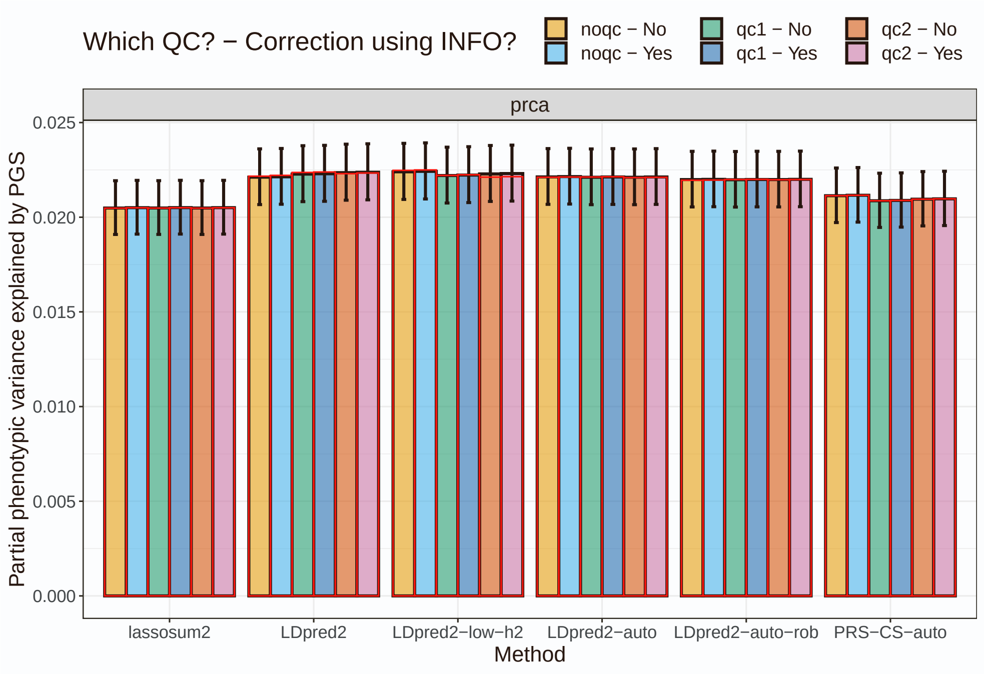

Figure S30: Variance explained of PRCA in the UK Biobank by PGS derived from external summary statistics. These are computed using function `pcor` of R package bigstatsr where 95% confidence intervals are obtained through Fisher's Z-transformation; these values are then squared.

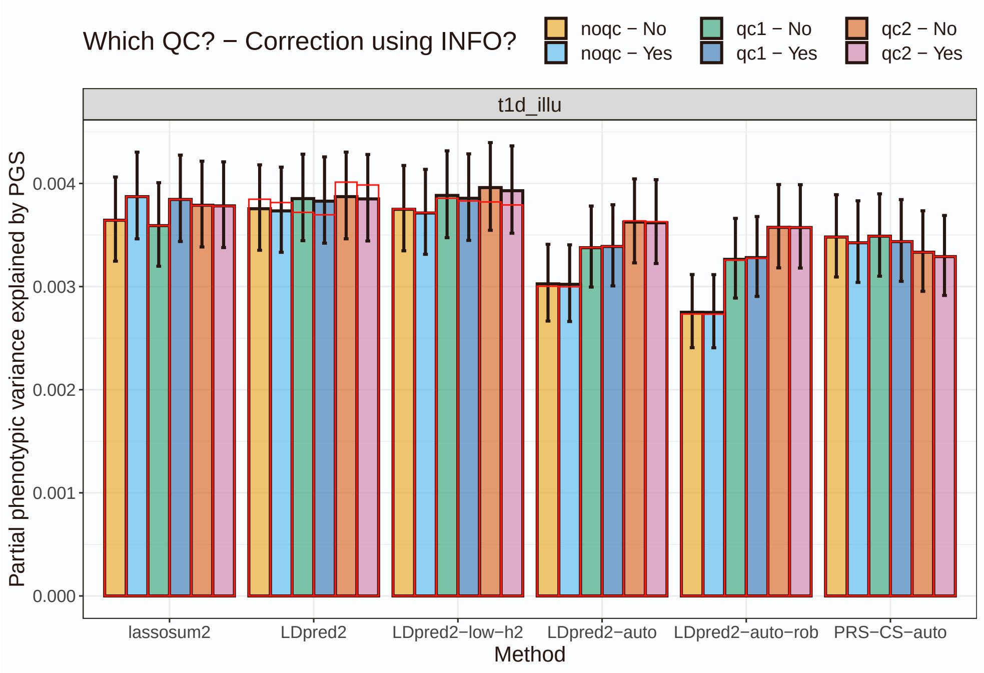

Figure S31: Variance explained of T1D in the UK Biobank by PGS derived from external summary statistics (Illumina). These are computed using function `pcor` of R package bigstatsr where 95% confidence intervals are obtained through Fisher's Z-transformation; these values are then squared.

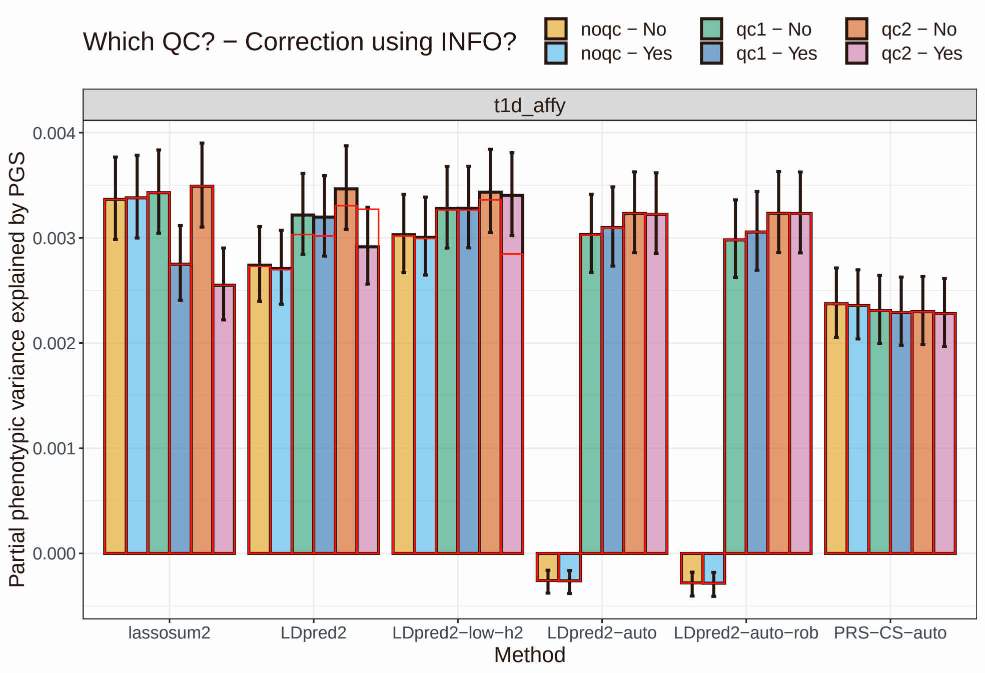

Figure S32: Variance explained of T1D in the UK Biobank by PGS derived from external summary statistics (Affymetrix). These are computed using function `pcor` of R package bigstatsr where 95% confidence intervals are obtained through Fisher's Z-transformation; these values are then squared (while keeping the sign).

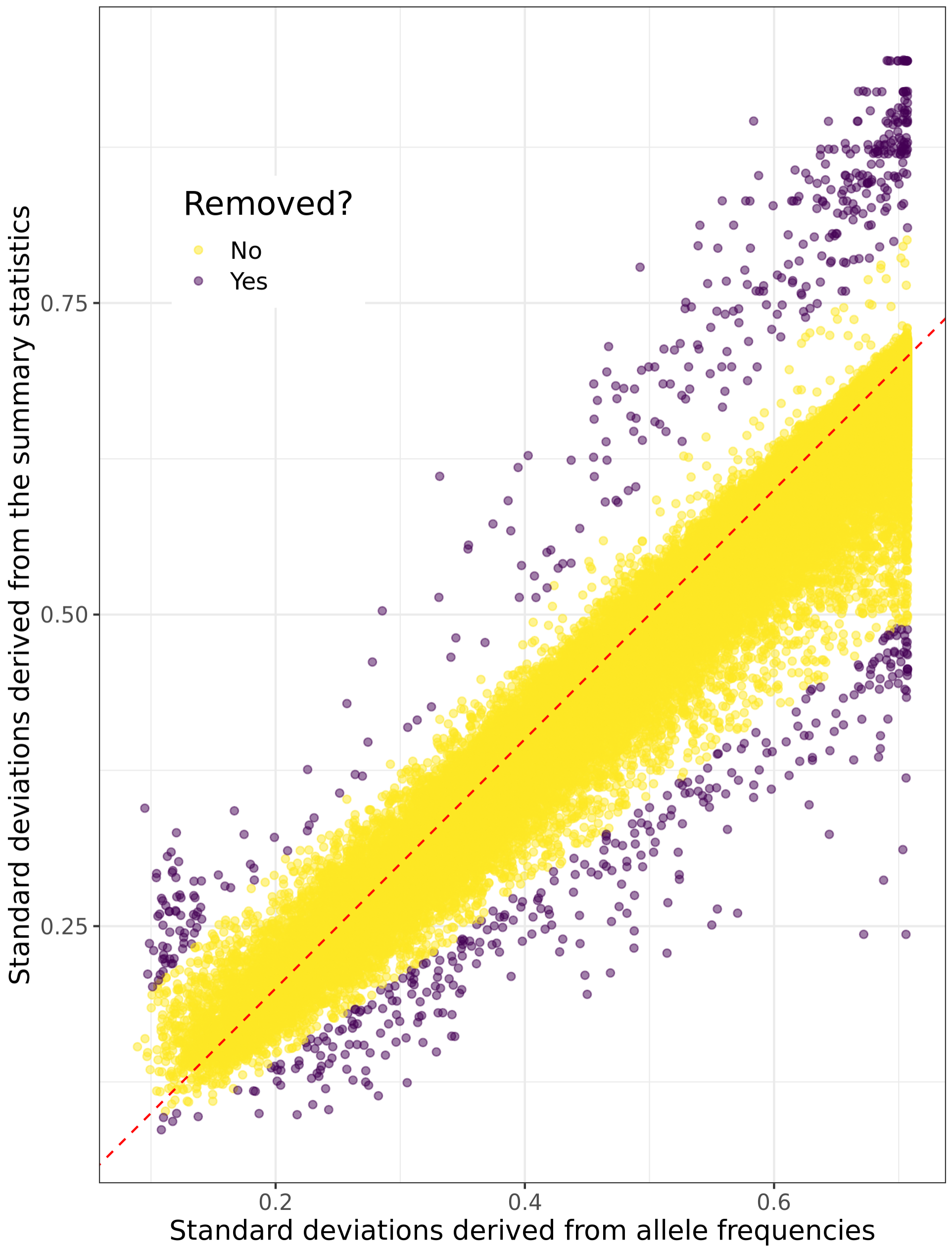

Figure S33: Standard deviations inferred from the vitamin D GWAS summary statistics versus the ones inferred from the allele frequencies of the LD reference. Only 100,000 variants are represented, at random.

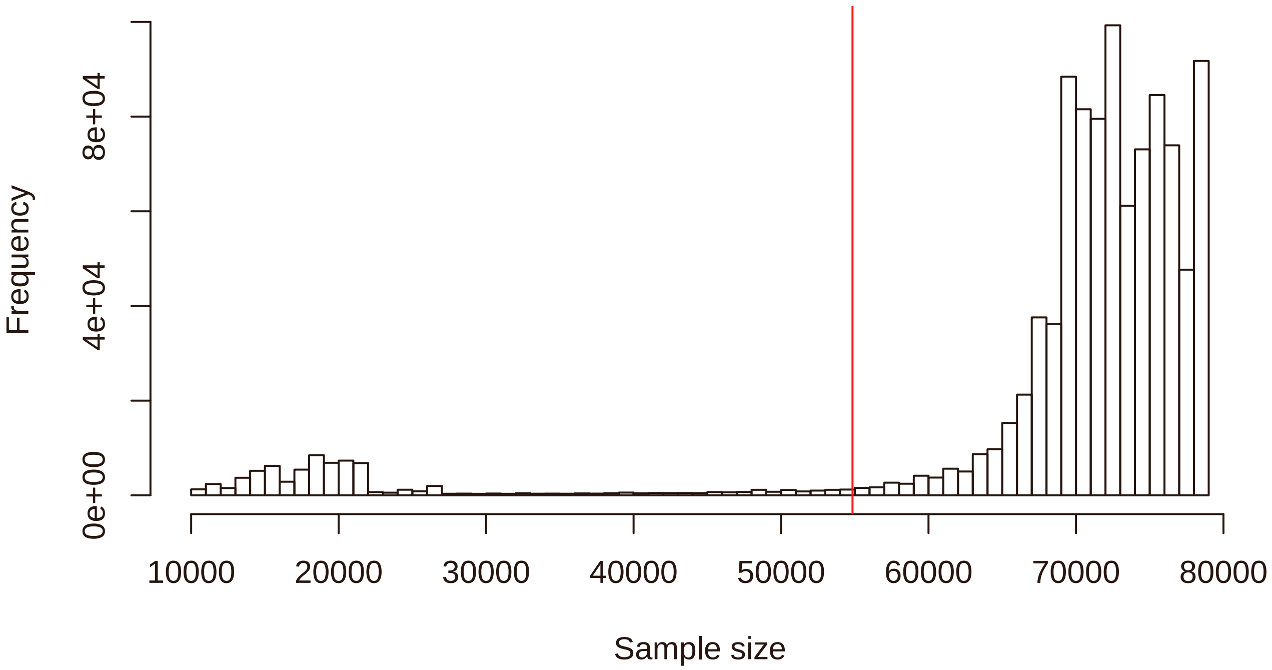

Figure S34: Histogram of the per-variant sample sizes in the vitamin D GWAS summary statistics. The vertical red line corresponds to 70% of the maximum sample size, the threshold used in “qc2”.

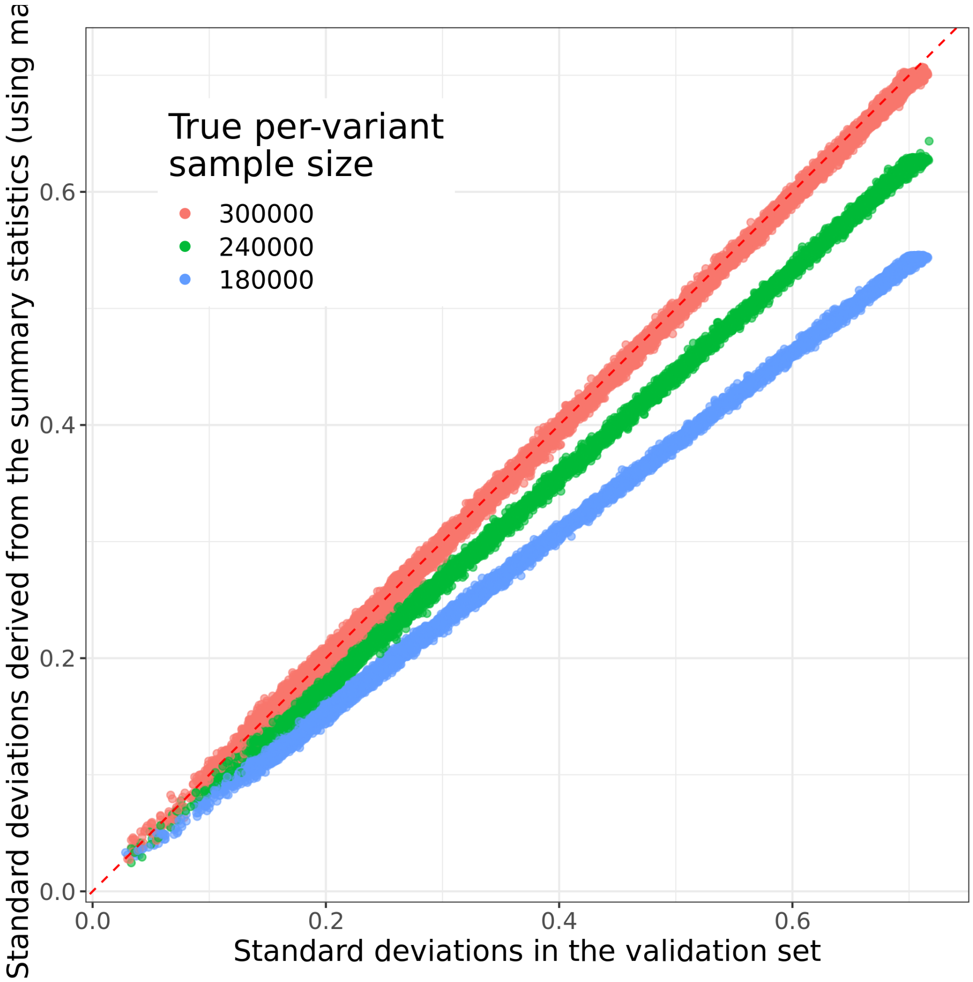

Figure S35: Quality control plot, as proposed in Privé et al.[6], for the simulations with sample size misspecification. The standard deviations are derived from the summary statistics assuming the same global GWAS sample size for all variants (300,000).

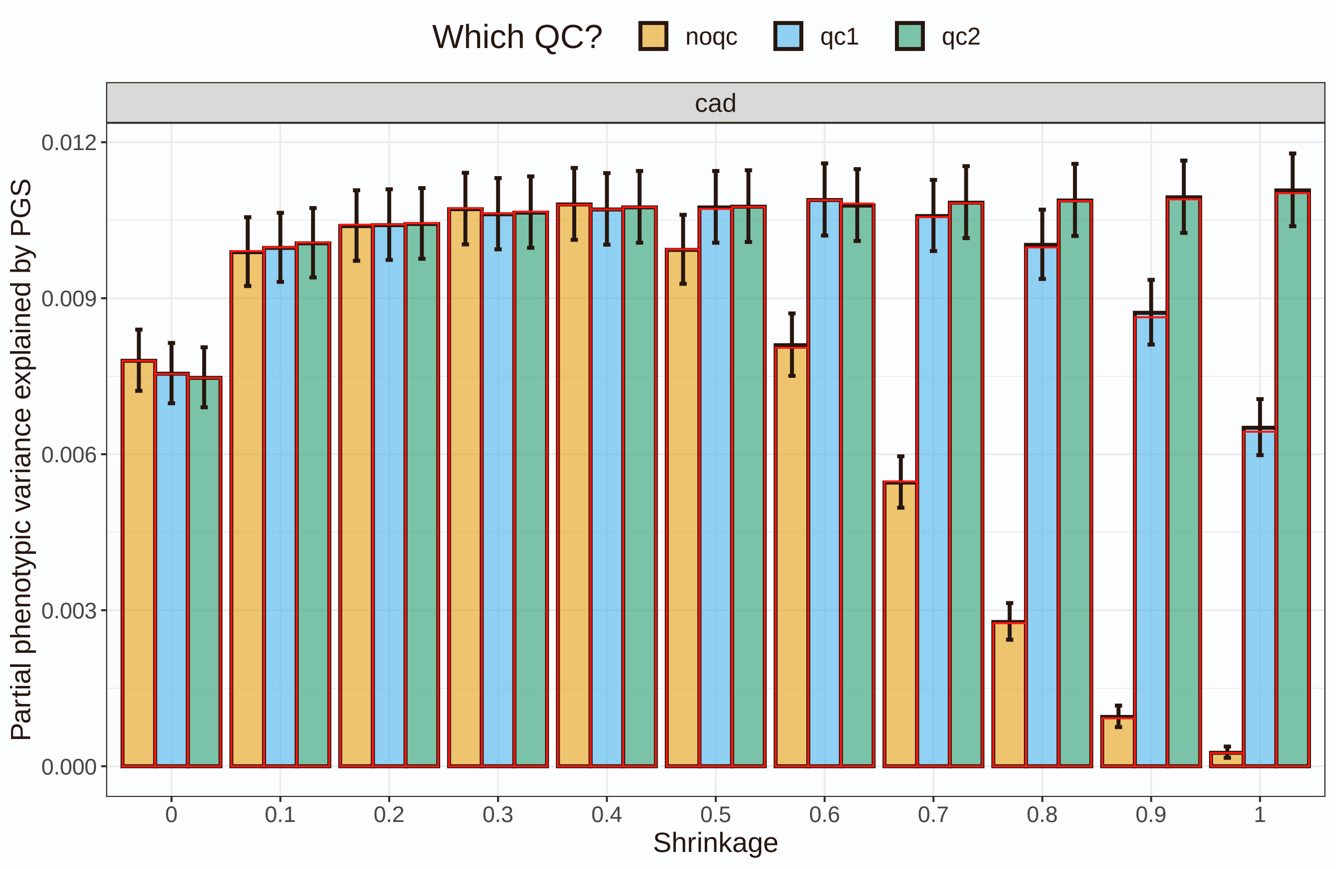

Figure S36: Variance explained of CAD in the UK Biobank by PGS derived from external summary statistics. These are computed using function `pcor` of R package bigstatsr where 95% confidence intervals are obtained through Fisher's Z-transformation; these values are then squared. Red bars correspond to using the LD with independent blocks (Methods). The shrinkage corresponds to the new parameter `shrink_corr` of LDpred2-auto.

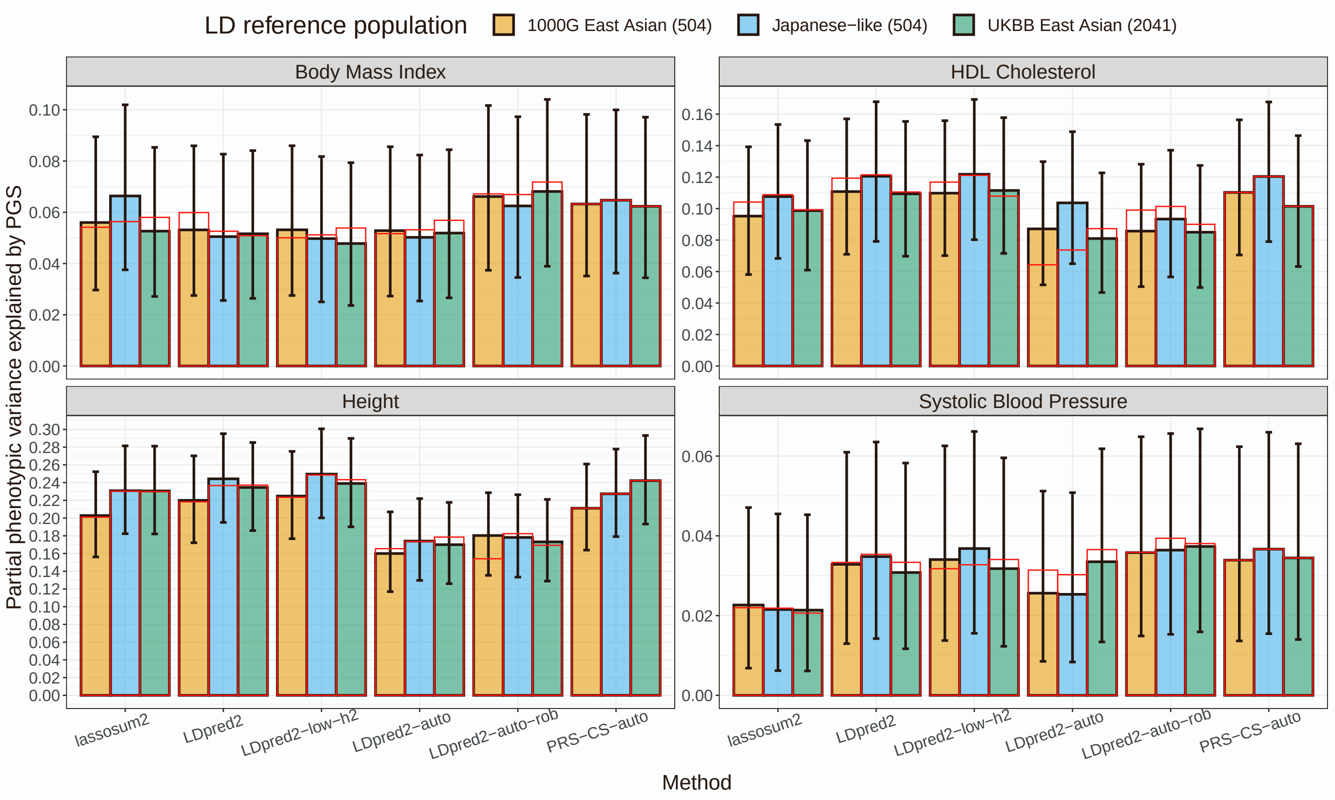

Figure S37: Results for PGS derived from four Biobank Japan GWAS summary statistics and using three different LD references. Partial correlations are computed using function `pcor` of R package bigstatsr where 95% confidence intervals are obtained through Fisher's Z-transformation, then all values are squared to report the phenotypic variance explained by PGS.

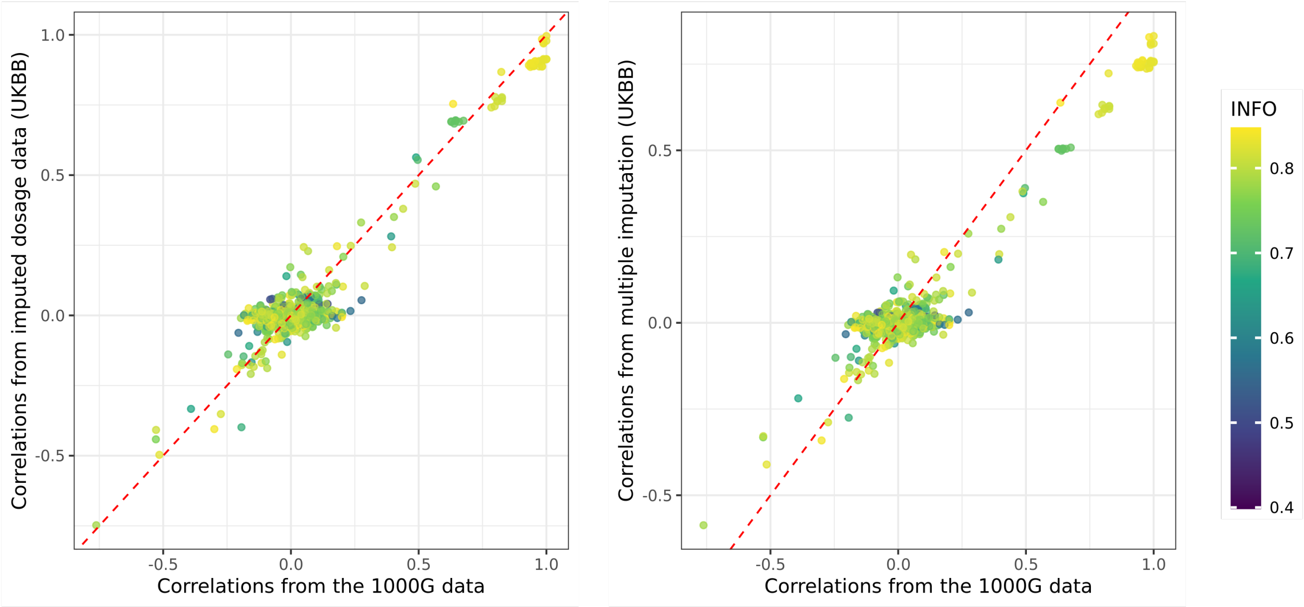

Figure S38: Comparing pairwise correlations between a subset of HapMap3 variants on chromosome 1 with an INFO score lower than 0.85, computed in three different ways: 1/ (left y-axis) from imputed dosages using 10,000 individuals from the UK Biobank (UKBB) data; 2/ (right y-axis) from multiple imputation (i.e. generating multiple complete datasets sampled according to imputation probabilities, computing correlations, and averaging results) also using UKBB; 3/ (common x-axis) from 190 individuals from the 1000 Genomes data (GBR and CEU). Each point, representing the correlation between two variants, is colored by the geometric mean of the INFO scores of these two variants.

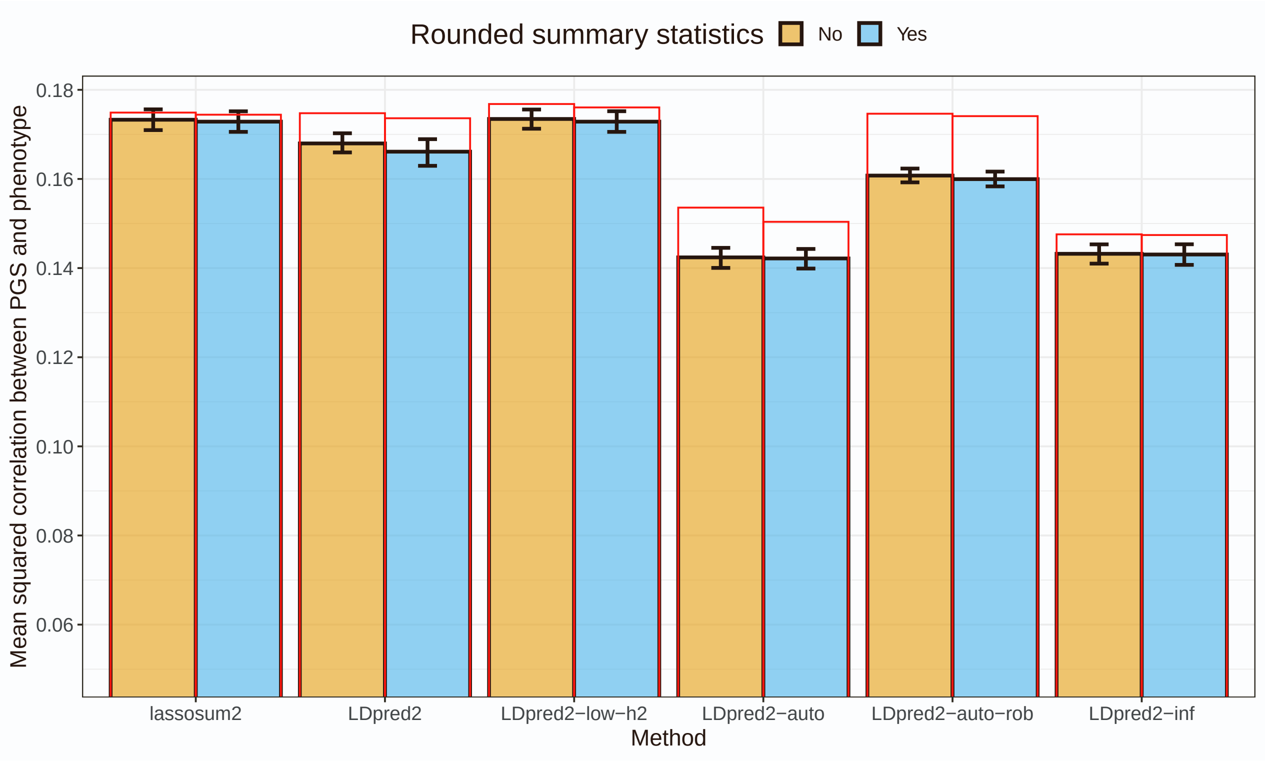

Figure S39: Results for the simulations with summary statistics (effect sizes and their standard errors) possibly rounded to two significant digits, averaged over 10 simulations for each scenario. Reported 95% confidence intervals are computed from 10,000 non-parametric bootstrap replicates of the mean. Red bars correspond to using the LD with independent blocks (Methods).

References

[1] Timothy Shin Heng Mak, Robert Milan Porsch, Shing Wan Choi, Xueya Zhou, and Pak Chung Sham. Polygenic scores via penalized regression on summary statistics. *Genetic Epidemiology*, 41(6):469–480, 2017.

[2] Luke R Lloyd-Jones, Jian Zeng, Julia Sidorenko, Loïc Yengo, Gerhard Moser, Kathryn E Kemper, Huanwei Wang, Zhili Zheng, Reedik Magi, Tonu Esko, et al. Improved polygenic prediction by Bayesian multiple regression on summary statistics. *Nature Communications*, 10(1):1–11, 2019.

[3] Cameron Palmer and Itsik Pe'er. Bias Characterization in Probabilistic Genotype Data and Improved Signal Detection with Multiple Imputation. *PLoS Genetics*, 12(6):e1006091, jun 2016. ISSN 15537404. doi: 10.1371/journal.pgen.1006091. URL `http://dx.plos.org/10.1371/journal.pgen.1006091`.

[4] Kyriaki Michailidou, Sara Lindström, Joe Dennis, Jonathan Beesley, Shirley Hui, Siddhartha Kar, Audrey Lemaçon, Penny Soucy, Dylan Glubb, Asha Rostamianfar, et al. Association analysis identifies 65 new breast cancer risk loci. *Nature*, 551 (7678):92–94, 2017.

[5] Kyriaki Michailidou, Per Hall, Anna Gonzalez-Neira, Maya Ghoussaini, Joe Dennis, Roger L Milne, Marjanka K Schmidt, Jenny Chang-Claude, Stig E Bojesen, Manjeet K Bolla, et al. Large-scale genotyping identifies 41 new loci associated with breast cancer risk. *Nature Genetics*, 45(4):353–361, 2013.

[6] Florian Privé, Julyan Arbel, and Bjarni J Vilhjálmsson. LDpred2: better, faster, stronger. *Bioinformatics*, 36(22-23): 5424–5431, 12 2020. ISSN 1367-4803. doi: 10.1093/bioinformatics/btaa1029. URL `https://doi.org/10.1093/bioinformatics/btaa1029`.
